# Polyadenylation Complex CFII Recognizes Downstream Cis‐element for Pre‐mRNA Polyadenylation Through Interaction with an RNA‐Binding Protein in Arabidopsis

**DOI:** 10.1002/advs.202504562

**Published:** 2025-08-11

**Authors:** Ying Cao, Ying Guo, Zhibo Yu, Huajian Nie, Jing Yang, Dingfu Qiu, Qiyu Li, Xu Xin, Chang Cheng, Yan Li, Xudong Shang, Yuling Jiao, Qingshun Quinn Li, Ligeng Ma

**Affiliations:** ^1^ College of Life Sciences Capital Normal University Beijing 100048 China; ^2^ Key Laboratory of the Ministry of Education for Coastal and Wetland Ecosystem, College of the Environment and Ecology Xiamen University Xiamen Fujian 361102 China; ^3^ Biomedical Sciences, College of Dental Medicine Western University of Health Sciences Pomona CA 91766 USA; ^4^ College of Life Sciences Peking University Beijing 100871 China

**Keywords:** accessory protein, alternative polyadenylation, CFII complex, downstream cis‐element, FPA, GA‐rich element, RNA‐binding protein

## Abstract

Alternative polyadenylation of pre‐mRNA generates mRNAs with alternative 3′ ends, thereby increasing the complexity of the transcriptome and proteome. This process is carried out by pre‐mRNA 3′ end processing complexes, with the specificity and site selection of polyadenylation primarily defined by the interaction between these complexes and conserved pre‐mRNA cis‐elements. However, the cis‐element recognized by the polyadenylation CFII complex and the downstream cis‐element of the poly(A) site remain unknown. Here, FPA, a conserved Spen family RNA‐binding protein, is identified to physically interacts with the CFII complex in *Arabidopsis*. FPA specifically binds to a GA‐rich cis‐element downstream of the proximal poly(A) site, which is required for the CFII complex to promote proximal poly(A) site usage and prevent 3′ extension. Thus, the CFII complex recognizes the downstream cis‐element of the poly(A) site through its interaction with FPA, highlighting the role of RNA‐binding proteins as accessory factors in alternative polyadenylation.

## Graphical abstract

This study investigates the cis‐element in pre‐mRNA recognized by the polyadenylation CFII complex. It elucidates how the CFII complex recognizes the consensus cis‐element downstream of the poly(A) site for pre‐mRNA alternative polyadenylation. This comprehensive research provides novel insights into the interaction between 3′ end processing complexes and pre‐mRNA, revealing mechanisms for polyadenylation site selection.

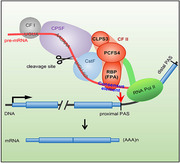

## 1 Introduction

The 3′ end of the vast majority of eukaryotic mature mRNAs contains a poly(A) tail, which is added co‐transcriptionally or post‐transcriptionally to the 3′ end of the transcript through a two‐step process: endonucleolytic cleavage of pre‐mRNA followed by poly(A) tail addition.^[^ ^1^, ^2^, ^3^, ^4^ ^]^ However, more than half of transcripts have multiple sites for 3′ end cleavage and polyadenylation, leading to alternative polyadenylation (APA) during RNA processing in mammals and higher plants.^[^ ^5^, ^6^, ^7^ ^]^ APA generates mRNA isoforms with alternative 3′ ends that differ in their coding sequences or 3′ untranslated regions (3′ UTRs), increasing the complexity of the transcriptome and proteome.^[^ ^2^, ^8^, ^9^, ^10^ ^]^ These isoforms may vary in function, stability, localization, and translation efficiency, and can affect protein subcellular localization. This widespread phenomenon across eukaryotes is a major mechanism of gene expression regulation.^[^ ^2^, ^8^, ^9^, ^10^ ^]^ APA occurs in a cell type‐ or tissue‐specific manner, is regulated by environmental cues, and is essential for cell proliferation, differentiation, and environmental response.^[^ ^5^, ^6^, ^11^, ^12^, ^13^, ^14^, ^15^, ^16^ ^]^ Dysfunction in APA is associated with diseases such as cancer and neurological disorders in humans, and developmental defects in plants.^[^ ^3^, ^7^, ^17^, ^18^, ^19^, ^20^ ^]^

Polyadenylation is executed by a highly conserved pre‐mRNA 3′ end processing machinery that comprises ≈80 proteins, of which ≈20 are core components conserved from yeast to plants and vertebrates.^[^ ^2^, ^3^, ^4^, ^21^ ^]^ This core machinery consists of four interconnected complexes: cleavage and polyadenylation factor (CPSF), cleavage stimulation factor (CstF), cleavage factor I (CFI) and II (CFII), as well as additional proteins such as poly(A) polymerase (PAP) and symplekin.^[^ ^21^, ^22^, ^23^, ^24^, ^25^ ^]^ The specificity and site selection of pre‐mRNA polyadenylation are primarily determined by a set of conserved sequence elements in the pre‐mRNA that are recognized by these complexes.^[^ ^4^, ^26^, ^27^ ^]^ These cis‐acting elements collectively define the functional poly(A) site, which is recognized by the poly(A) machinery to execute the cleavage and polyadenylation reaction. In metazoans, the CPSF complex recognizes the conserved poly(A) site core hexamer element AAUAAA (or its variants), the CFI complex recognizes the UGUA element upstream of AAUAAA, and the CstF complex recognizes the U‐rich or GU‐rich element downstream of AAUAAA.^[^ ^4^, ^26^, ^27^ ^]^ In higher plants, three cis‐elements‐near upstream element (NUE), far‐upstream element (FUE), and cleavage element (CE)‐are functionally equivalent to AAUAAA, UGUA, and U‐rich (or GU‐rich) elements or their variants, respectively.^[^ ^28^, ^29^, ^30^, ^31^, ^32^ ^]^ The recognition between complexes and specific consensus elements is achieved through direct binding between the consensus element and an RNA‐binding protein within each complex. This binding is critical for recruiting additional cleavage and polyadenylation factors to form the functional machinery at the poly(A) site, thereby promoting 3′ end processing.^[^ ^21^, ^22^, ^23^, ^24^, ^25^, ^33^, ^34^ ^]^ However, despite the essential role of the CFII complex in alternative polyadenylation, no RNA‐binding protein has been identified within it, and no specific sequence elements around the poly(A) site have been shown to interact with CFII.^[^ ^4^, ^7^, ^20^, ^34^ ^]^ Consequently, the mechanism by which the CFII complex recognizes the poly(A) site to perform cleavage and polyadenylation in eukaryotes remains unknown. Additionally, no downstream cis‐element of the poly(A) site required for efficient cleavage and polyadenylation in plants has been identified to date.^[^ ^28^, ^29^, ^30^, ^31^, ^32^ ^]^

*FPA* was initially identified as an activator of the floral transition in *Arabidopsis*, functioning in the autonomous pathway by repressing *FLC* expression.^[^ ^35^, ^36^ ^]^ It encodes a Spen family protein with three RNA‐recognition motif (RRM) domains in its N‐terminal region, suggesting potential RNA‐binding activity.^[^ ^37^ ^]^ Mutations in *FPA* alter alternative polyadenylation patterns in its own pre‐mRNA, antisense *FLC* transcripts, *NLR* genes, and several others, and cause transcript read‐through and 3′ extension in *Arabidopsis*.^[^ ^38^, ^39^, ^40^, ^41^, ^42^, ^43^, ^44^ ^]^ These findings indicate that FPA is crucial for alternative polyadenylation, although the underlying molecular mechanism remains unclear.

In this study, we demonstrate that FPA physically interacts with the polyadenylation CFII complex and is essential for its function in *Arabidopsis*. The CFII complex promotes proximal poly(A) site usage and prevents 3′ extension by binding to a GA‐rich element downstream of the poly(A) site. This element is distinct from the three other cis‐elements recognized by CPSF, CFI, and CstF complexes. Our results show that FPA‐dependent recognition of this GA‐rich element by the CFII complex specifies pre‐mRNA species for cleavage and polyadenylation. Thus, our work not only elucidates the mechanism of FPA‐mediated alternative polyadenylation in *Arabidopsis* but also identifies the downstream cis‐element of the poly(A) site. Furthermore, it reveals that the CFII complex recognizes a consensus element through interaction with an RNA‐binding protein, acting as an accessory factor to sort pre‐mRNAs for processing.

## 2 Results

### 2.1 CFII Complex Physically Interacts and Closely Associates with an RNA‐Binding Protein FPA

**Figure 1:**
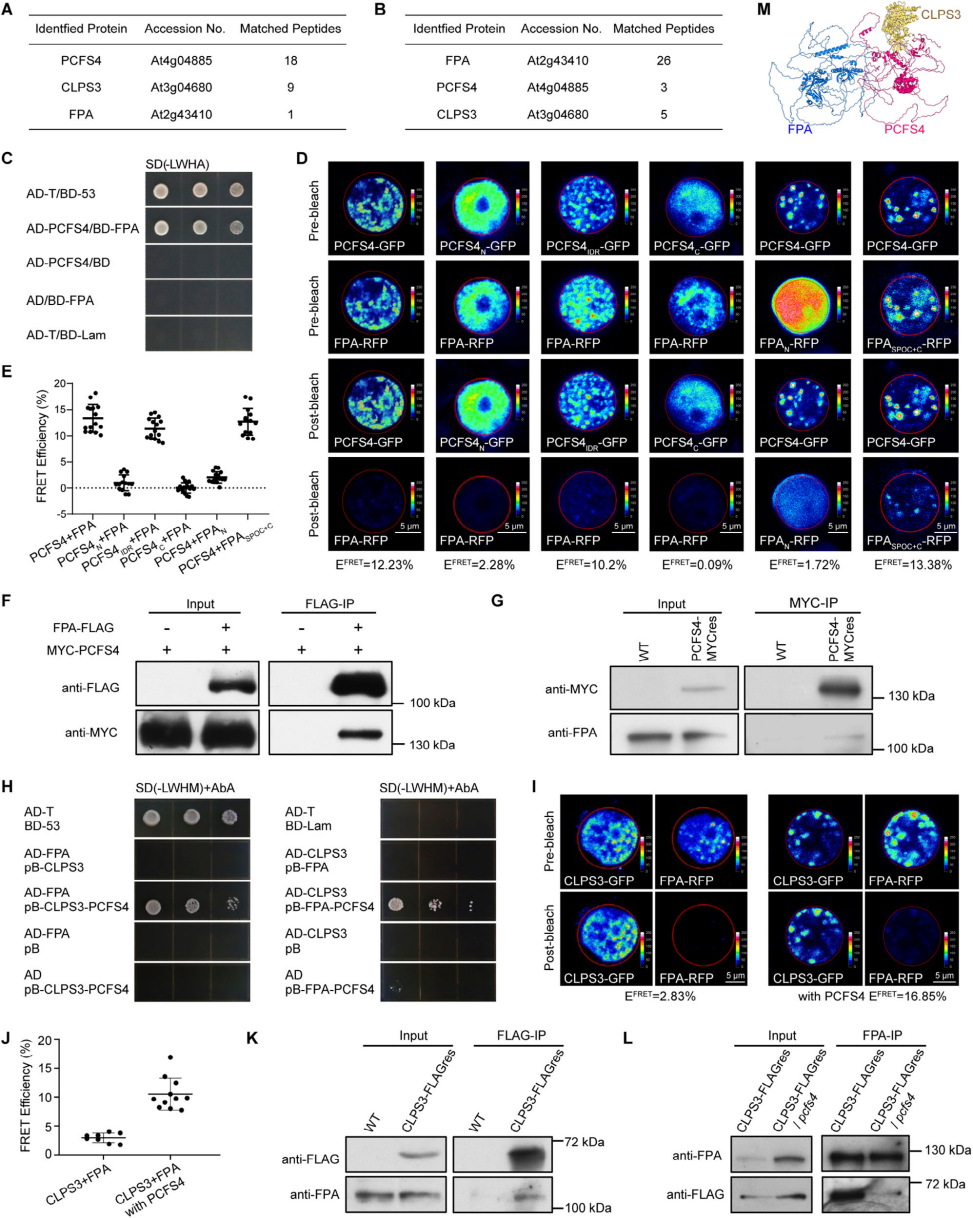
FPA physically interacts with CFII complex in *Arabidopsis* A) PCFS4 co‐purified with CLPS3 and FPA identified by PCFS4‐MYC affinity purification. PCFS4‐interacting proteins were identified by co‐immunoprecipitation using anti‐Myc magnetic beads in PCFS4‐MYC transgenic plants, followed by LC‐MS/MS analysis. Wild‐type plants were used as a negative control. For detailed information, see Table S1 (Supporting Information). B) FPA co‐purified with PCFS4 and CLPS3 identified by FPA‐MYC affinity purification. FPA‐interacting proteins were identified by co‐immunoprecipitation using anti‐Myc magnetic beads in FPA‐MYC transgenic plants, followed by LC‐MS/MS analysis. Wild‐type plants were used as a negative control. Detailed information is provided in Table S2 (Supporting Information). C) Yeast two‐hybrid assay to demonstrate the direct interaction between FPA and PCFS4. Positive control: AD‐T/BD‐53; negative control: AD‐T/BD‐Lam. The two indicated plasmids were co‐transformed into the yeast reporter strain AH109. The interaction between the two tested proteins was assessed by growth on SD/‐LWHA medium. D) Acceptor photobleaching FRET assay to demonstrate the interaction between FPA and PCFS4 in *N. benthamiana* cells. Column 1 shows the interaction between full‐length PCFS4 and full‐length FPA; Columns 2, 3, and 4 depict the interactions between each of the three truncated PCFS4 proteins (PCFS4_N_/_IDR_/_C_) and full‐length FPA; Columns 5 and 6 illustrate the interactions between full‐length PCFS4 and each of the two truncated FPA proteins (FPA_N_/FPA_SPOC+C_, See Figure S1B,C (Supporting Information) for schematic diagrams). GFP (donor) and RFP (acceptor) fluorescence intensities were measured before and after acceptor photobleaching. The FRET efficiency was calculated as E_FRET_ = (I_Doner_^post^ – I_Doner_^pre^)/I_Doner_^post^. E) Summary of FRET efficiencies for the interaction between FPA and PCFS4. F) Co‐immunoprecipitation (Co‐IP) assay to detect the physical interaction between FPA and PCFS4 in *Arabidopsis* protoplasts. The indicated proteins were expressed in *Arabidopsis* protoplasts, immunoprecipitated with M2 magnetic beads, and probed with the corresponding antibodies. G) Co‐IP assay to detect the physical interaction between FPA and PCFS4 in *Arabidopsis* plants. Total protein extracts from wild‐type (WT) or PCFS4‐MYC plants were immunoprecipitated using anti‐Myc magnetic beads and subsequently probed with the indicated antibodies. H) Yeast three‐hybrid assay to demonstrate the interaction between FPA and CLPS3 via PCFS4. Positive control: AD‐T/BD‐53; negative control: AD‐T/BD‐Lam. Transformed Y2H Gold yeast cells were plated on SD/‐LWHM+AbA medium to detect protein‐protein interactions mediated by the indicated plasmid combinations. I) Acceptor photobleaching FRET assay to demonstrate the interaction between FPA and CLPS3 via PCFS4 in *N. benthamiana* cells. The interactions between FPA and CLPS3, with or without PCFS4, were assessed. GFP (donor) and RFP (acceptor) fluorescence intensities were measured before and after acceptor photobleaching. The FRET efficiency was calculated as E_FRET_ = (I_Doner_^post^ – I_Doner_^pre^)/I_Doner_^post^. J) Summary of FRET efficiencies between FPA and CLPS3 with or without the presence of PCFS4. K) Co‐IP assay to detect the physical interaction between FPA and CLPS3 in *Arabidopsis* plants. Total protein extracts from wild‐type (WT) or CLPS3‐FLAG plants were immunoprecipitated using anti‐FLAG M2 magnetic beads and subsequently probed with the indicated antibodies. L) Co‐IP assay to demonstrate the dependence of PCFS4 for the interaction between FPA and CLPS3 in *Arabidopsis* plants. Total protein extracts from CLPS3‐FLAG plants and CLPS3‐FLAG/*pcfs4* plants were immunoprecipitated using anti‐FPA antibodies and subsequently probed with the indicated antibodies. M) Schematic diagram to show that PCFS4 bridges the interaction between FPA and CLPS3. The structures of three proteins and the docking of their interaction were predicted by AlphaFold2. See Figure S3F (Supporting Information) for an enlarged image. Also see Figures S1,S2, and S3 (Supporting Information).

PCFS4 and CLPS3 are homologs of mammalian Pcf11 and Clp1 in *Arabidopsis*, respectively, and are the only known components of the CFII complex in eukaryotes.^[^ ^45^, ^46^ ^]^ Given that the other three polyadenylation complexes (CPSF, CFI, and CstF) each include an RNA‐binding protein to recognize cis‐elements in pre‐mRNA,^[^ ^28^, ^31^, ^32^, ^47^, ^48^ ^]^ we investigated whether additional RNA‐binding proteins interact with the CFII complex. To this end, we performed IP‐MS (immunoprecipitation‐mass spectrometry) assays to identify PCFS4‐interacting proteins in *Arabidopsis*. The PCFS4‐MYC affinity purification analysis revealed that PCFS4 interacts with FPA, CLPS3, and several other components of the 3′ cleavage and polyadenylation complex (**Figure** **1**A; Table S1, Supporting Information). This suggests that PCFS4 may interact with an RNA‐binding protein.

To further examine FPA's involvement in the polyadenylation machinery, we conducted IP‐MS assays to identify FPA‐interacting proteins. The FPA‐MYC affinity purification analysis also identified interactions with PCFS4, CLPS3, and other components of the mRNA cleavage and polyadenylation complex (Figure 1B; Table S2, Supporting Information). These results indicate that the CFII complex and/or other polyadenylation machinery complexes may physically interact with the RNA‐binding protein FPA in *Arabidopsis*.

To confirm this interaction, we performed preliminary Co‐IP assays between FPA and several components of the polyadenylation complexes in *Arabidopsis* protoplasts. We observed that FPA physically interacts with PCFS4 but not with CPSF30, CPSF100 (components of the CPSF complex), or CstF64 (a component of the CstF complex) (Figure S1A, Supporting Information). This suggests that FPA is more closely associated with PCFS4 in *Arabidopsis*.

We further confirmed the physical interaction between FPA and PCFS4 using yeast two‐hybrid assays, FRET in tobacco cells, and Co‐IP assays in both *Arabidopsis* protoplasts and whole plants (Figure 1C–G). Additionally, we found that the SPOC domain of FPA, a conserved domain among Spen family proteins in eukaryotes, is required for FPA to interact with PCFS4. Conversely, the intrinsically disordered region (IDR) of PCFS4 is required for PCFS4 to interact with FPA (Figure 1D,E; Figures S1B,C, and S2, Supporting Information). These results demonstrate that PCFS4 physically interacts with FPA and highlight the domains involved in this interaction.

We next examined whether FPA interacts with the polyadenylation CFII complex in *Arabidopsis*. Since PCFS4 and CLPS3 are homologs of mammalian CFII components Pcf11 and Clp1, respectively,^[^ ^45^, ^46^ ^]^ we first tested whether PCFS4 and CLPS3 form a complex in *Arabidopsis*. We verified their interaction in yeast and found that the C‐terminal domain of PCFS4 is required for its interaction with CLPS3 (Figure S3A, Supporting Information). This interaction was further confirmed by FRET assays in tobacco cells and Co‐IP assays in *Arabidopsis* (Figure S3B,C, Supporting Information). These results indicate that, similar to their counterparts in yeast and animals,^[^ ^49^ ^]^ PCFS4 and CLPS3 form a complex in *Arabidopsis*. Having confirmed the physical interactions between FPA and PCFS4, and between PCFS4 and CLPS3, we next investigated whether FPA physically interacts with CLPS3. Yeast two‐hybrid assays showed no direct interaction between FPA and CLPS3 (Figure S3D, Supporting Information). However, a yeast three‐hybrid assay in the presence of PCFS4 revealed an interaction between FPA and CLPS3 (Figure 1H). This observation was further supported by FRET assays, which showed no FRET signal between FPA and CLPS3 in the absence of PCFS4, but a strong FRET signal in its presence (Figure 1I,J). Co‐IP assays in *Arabidopsis* plants demonstrated that FPA and CLPS3 are in the same complex, and their interaction was significantly reduced in the *pcfs4* mutant (Figure 1K,L; Figure S3E, Supporting Information). These results indicate that FPA physically interacts with the CFII complex, with PCFS4 acting as a bridge to facilitate the interaction between FPA and CLPS3 in *Arabidopsis* (Figure 1M; Figure S3F, Supporting Information).

### 2.2 Both FPA and PCFS4 Promote a Similar Set of Proximal Poly(A) Site Usage

**Figure 2:**
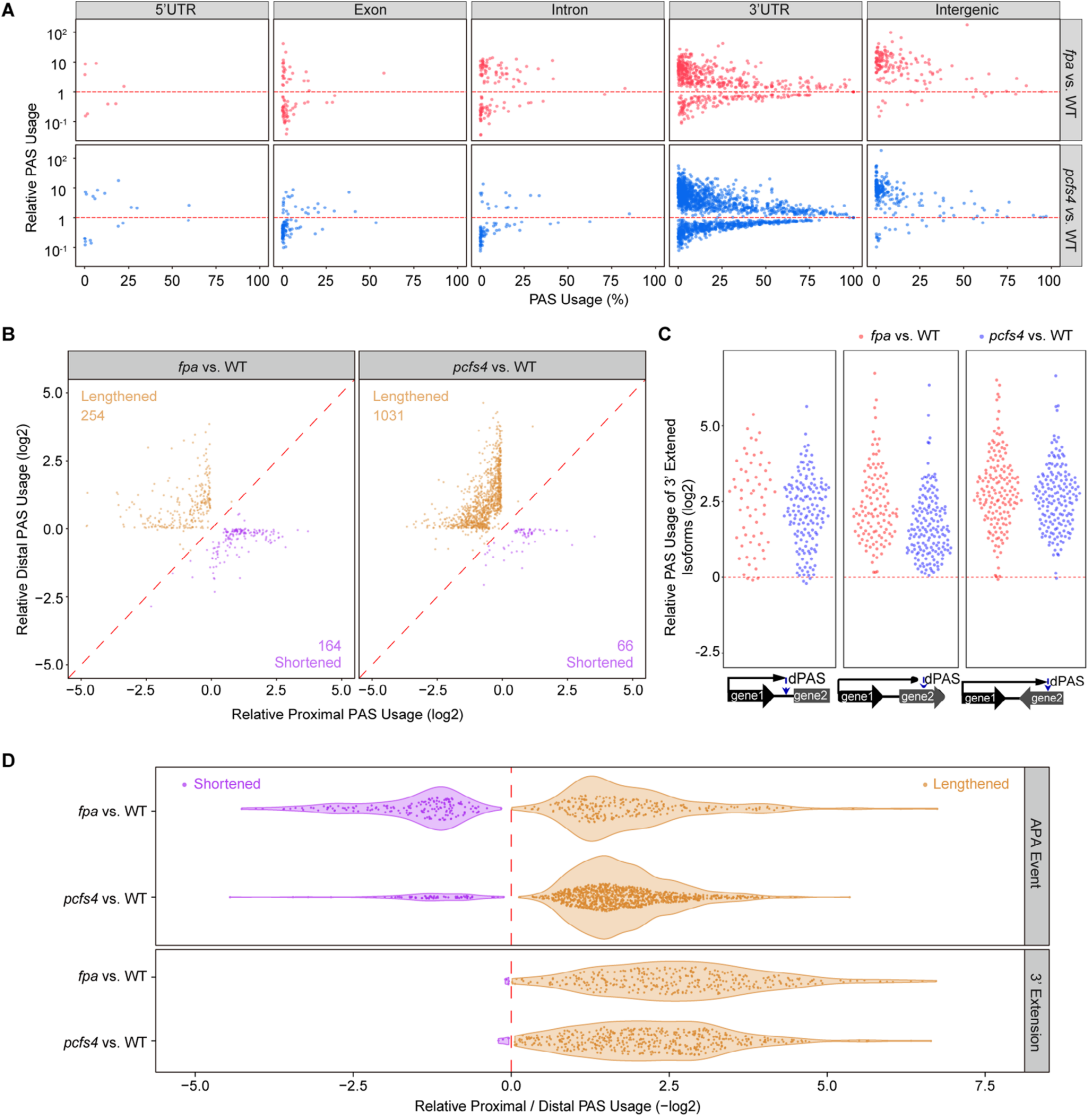
FPA and PCFS4 promote proximal poly(A) site usage A) Relative PAS usage for DEPASs from *fpa* versus WT and *pcfs4* versus WT. The *x*‐axis represents PAS usage for each DEPAS in WT, and the *y*‐axis represents the fold change of PAS usage in mutant relative to that of wild type, DEPAS in different regions of the gene transcript were analyzed. Each dot represents a DEPAS. B) APA events from *fpa* versus WT and *pcfs4* versus WT. The *x*‐axis shows the relative usage of proximal poly(A) sites on a log2 scale, while the *y*‐axis shows the relative usage of distal poly(A) sites on a log2 scale. Each dot represents an APA event, and the total number is shown for lengthened or shortened transcripts. C) 3′ extension events from *fpa* versus WT and *pcfs4* versus WT. 3′ extension events that stopped in the intergenic region (type 1) and the downstream gene (types 2 and 3) are analyzed. The *y*‐axis represents the relative usage of the distal poly(A) site of the 3′ extension isoform in the mutant, relative to the wild type (WT). D) Relative usages of proximal and distal PAS for the APA events from *fpa* versus WT and *pcfs4* versus WT. Each dot represents an APA event. Lengthened (shift to distal PAS) and shortened (shift to proximal PAS) APA events are shown in purple and brown, respectively. PAS usage = reads of one PAS isoform / total reads of the mRNA; Relative PAS usage = PAS usage (mutant) / PAS usage (WT). Lengthened (shift to distal PAS) and shortened (shift to proximal PAS) transcripts or 3′ UTRs in mutant versus WT in B–D exhibited a statistically significant deviation from total APA events, as determined by Kolmogorov–Smirnov test (K‐S test, *p*‐value < 0.001). pPAS: proximal poly(A) site, dPAS: distal poly(A) site. Also see Figure S8 (Supporting Information).

Given that the CFII complex is essential for 3′ cleavage and polyadenylation in both mammals and plants,^[^ ^45^, ^46^, ^50^ ^]^ and FPA has been reported to regulate these processes in *Arabidopsis*,^[^ ^38^, ^39^, ^40^, ^41^, ^42^, ^43^, ^44^ ^]^ we examined the genome‐wide polyadenylation landscape mediated by the CFII complex and FPA using Poly(A)‐Tag Sequencing (PAT‐Seq). Since mutations in *CLPS3* result in embryonic lethality in *Arabidopsis* (Figure S4A, Supporting Information),^[^ ^46^ ^]^ we focused on the T‐DNA insertion mutants of *fpa* and *pcfs4* (Figure S4B, Supporting Information) and analyzed the polyadenylation profiles of gene transcripts from wild‐type (WT), *fpa*, and *pcfs4* plants. PAT‐Seq data demonstrated high reproducibility among the three independent biological replicates (Figure S5A, Supporting Information). We identified ≈35000 poly(A) sites (PASs) in nine‐day‐old *Arabidopsis* plants (34977 for WT, 34179 for *fpa*, and 35748 for *pcfs4*). On average, there were ≈1.5 PASs per pre‐mRNA, with most PASs located in the 3′ UTR. Other PASs were found in intergenic regions, exons, introns, and 5′ UTRs, in descending order of frequency, and there is no global impairment of 3′ end polyadenylation in either *fpa* or *pcfs4* mutants (Figure S5B,C, Supporting Information). We identified differentially expressed PASs (DEPAS) that were not caused by transcriptional level changes between *fpa* or *pcfs4* and WT, using a threshold of fold change > 1.5 and *p* < 0.05 (Figure S6A–D, Supporting Information). It was observed that the DEPAS identified from the comparison of *fpa* and WT in this study exhibited a similar regulatory pattern to the DEPAS profiled by direct RNA sequencing in the *fpa‐8* versus WT, as previously published (Figure S7, Supporting Information). This consistency confirms the robustness of our findings. There were 1618 DEPAS in *fpa* versus WT and 3074 DEPAS in *pcfs4* versus WT (Table S3 and Figure S6A–D, Supporting Information). Most of these DEPAS were located in the 3′ UTR, with others in intergenic, exon, intron, and 5′ UTR regions (**Figure** **2**A; Figure S8A, Supporting Information). Overall, mutations in *FPA* and *PCFS4* led to decreased proximal poly(A) site usage and increased distal poly(A) site usage (Figure S8B, Supporting Information). This indicates that both FPA and PCFS4 generally promote proximal poly(A) site usage, thereby favoring the formation of shorter transcript isoforms.

To further analyze alternative polyadenylation events mediated by FPA and PCFS4 in *Arabidopsis*, we classified these events into two categories: APA events and 3′ extension (Figure S8C, Supporting Information). We observed 744 alternative polyadenylation events between WT and *fpa* plants, comprising 418 APA events and 326 3′ extension events. In contrast, there were 1551 alternative polyadenylation events between WT and *pcfs4* plants, including 1097 APA events and 454 3′ extension events (Table S4, Supporting Information). Further analysis revealed that mutations in *FPA* or *PCFS4* led to a similar defect in alternative polyadenylation, with more mRNAs showing a shift in poly(A) site usage from proximal to distal. This shift generally increased the usage of distal PASs and lengthened transcripts genome‐wide for those alternative polyadenylation events (Figure 2B–D). These findings indicate that both FPA and PCFS4 promote proximal poly(A) site usage and prevent 3′ extension, supporting the notion that FPA and PCFS4 function in the same polyadenylation complex to mediate alternative polyadenylation genome‐wide.

**Figure 3:**
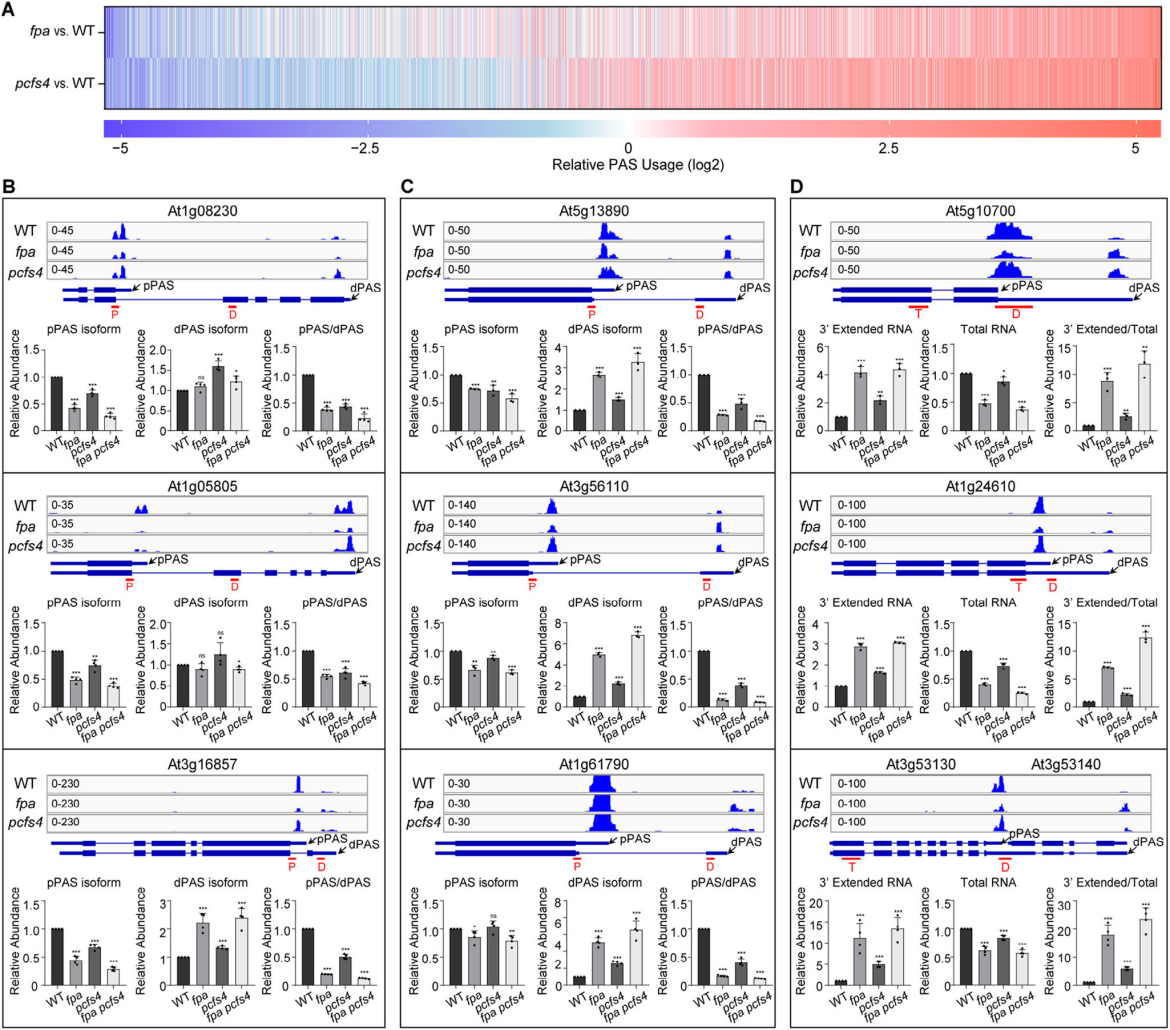
FPA and PCFS4 regulate 3′ APA events for a similar set of pre‐mRNAs A) Heat map of DEPAS in *fpa* versus WT and *pcfs4* versus WT. The color scale indicates the fold change in relative PAS usage, with red for increased usage and blue for decreased usage in the mutant versus WT. B‐D) PAT‐Seq and RT‐qPCR analyses from nine representative gene transcripts for APA events B,C) and 3′ extension D) in WT, *fpa*, and *pcfs4*. PAT‐Seq data are shown in the Integrative Genomics Viewer on the upper panel. For RT‐qPCR assays, the relative RNA abundance in *fpa*, *pcsf4* or *fpa pcsf4* was normalized to that in WT. Data are presented as Mean + SD, n = 3–4 biologically independent samples. Two‐tailed Student's *t*‐test was used to analyze the difference between the mutant and WT, ^***^ *p* < 0.001, ^**^ *p* < 0.01, ^*^ *p* < 0.05. The red lines under the pre‐mRNA structure represent the positions to detect APA isoforms. P and D were used to detect the pPAS and dPAS isoforms, respectively, in (B) and (C), while T and D were used to detect the total mRNA and 3′ extended isoform in (D), respectively. Primer sequences are provided in Table S5 (Supporting Information). pPAS: proximal poly(A) site, dPAS: distal poly(A) site. Also see Figure S8 (Supporting Information).

Heat map analysis of DEPAS mediated by FPA and PCFS4 revealed a similar regulatory pattern, suggesting that FPA and PCFS4 regulate a similar set of poly(A) sites in the *Arabidopsis* genome (**Figure** **3**A). We randomly selected nine representative APA and 3′ extension events regulated by both FPA and PCFS4 and analyzed the alternative polyadenylation patterns of the corresponding gene transcripts in *fpa*, *pcfs4* single, and *fpa pcfs4* double mutants using RT‐qPCR. Consistent with the genome‐wide analysis, mutations in *FPA* or *PCFS4* led to decreased proximal PAS usage, increased distal PAS usage, or 3′ extension in the mutants for these representative genes (Figure 3B–D). These results support the conclusion that FPA physically interacts with the CFII complex (via PCFS4) to regulate alternative polyadenylation and promote proximal poly(A) site usage of a similar set of mRNAs.

### 2.3 FPA Binds Downstream of the Pre‐mRNA Proximal Poly(A) Site and Promotes the Poly(A) Site Usage

Given that FPA is a predicted RNA‐binding protein,^[^ ^37^ ^]^ and our results suggest that it physically associates with the CFII complex, we investigated whether FPA binds to pre‐mRNAs. To this end, we transformed GFP‐tagged full‐length FPA (FPA‐GFP) into the *fpa* mutant background and observed that the transformation fully complemented the flowering phenotype of *fpa* (Figure S4C, Supporting Information). This indicates that FPA‐GFP is functional in *Arabidopsis*.

We then optimized the RNA immunoprecipitation sequencing (RIP‐Seq) assay by extending the sonication time after nuclei extraction to enhance nuclear protein extraction efficiency and reduce RNA fragment size. The resulting immunoprecipitated RNA fragments peaked ≈200 bp, and fragments between 100 and 300 bp were used for strand‐specific RNA sequencing (Figure S9A, Supporting Information). The RIP‐Seq analysis demonstrated high reproducibility among three independent biological replicates (Figure S9B, Supporting Information). We identified 3840 FPA binding sites corresponding to 3017 pre‐mRNA species in the *Arabidopsis* genome. Among these, 427 pre‐mRNAs exhibited differential expression of poly(A) sites (DEPAS) between WT and *fpa*.

**Figure 4:**
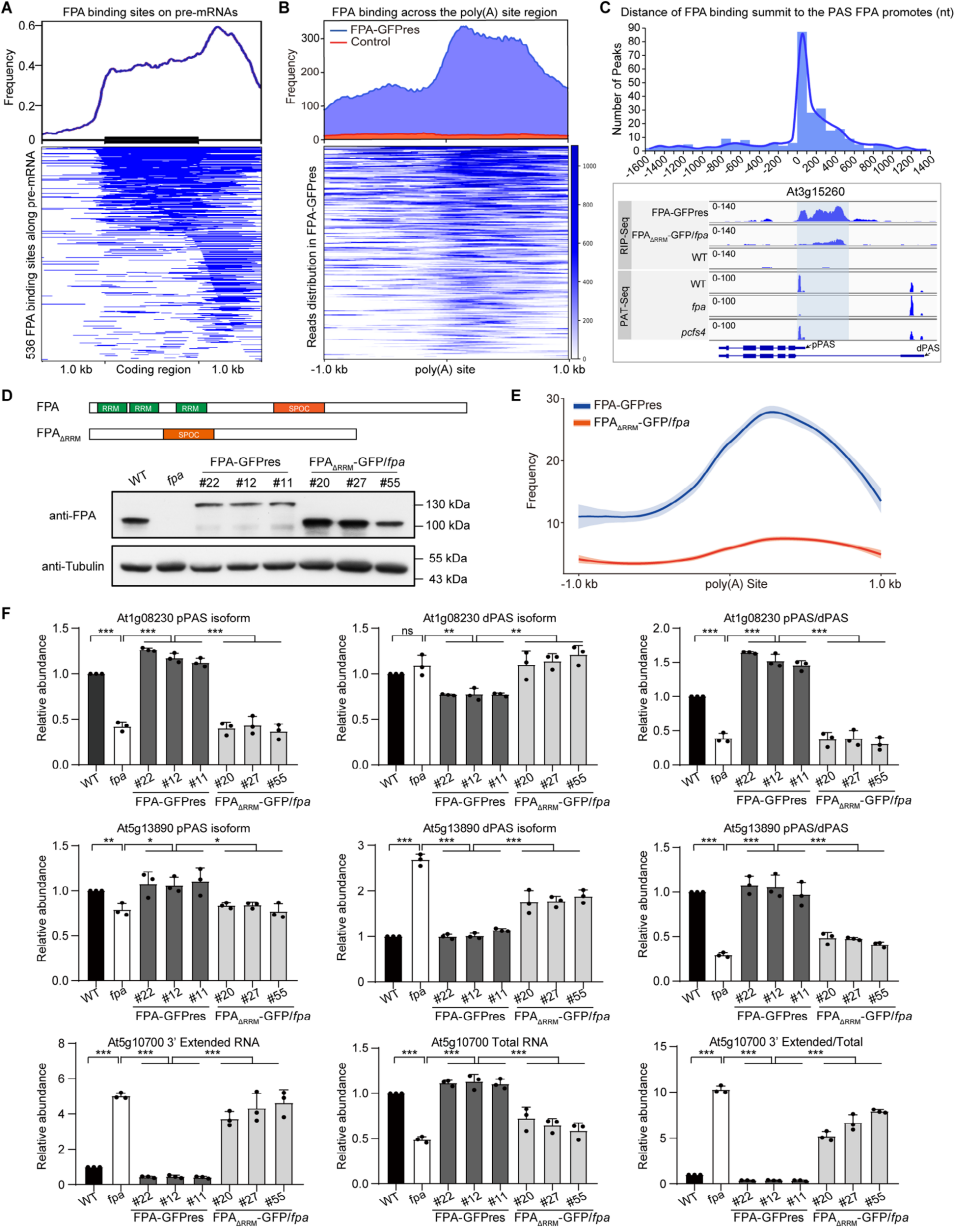
FPA binds downstream of the pre‐mRNA proximal poly(A) site and promotes the proximal poly(A) site usage A) Distribution of FPA binding sites in pre‐mRNA regions. 536 FPA binding peaks identified by MACS2 in FPA‐GFPres versus WT corresponding to 427 FPA‐mediated pre‐mRNAs with DEPAS were analyzed. B) FPA binds to the downstream region of PAS it promotes. Control: RIP‐Seq from WT. Reads distribution across the poly(A) site region was analyzed. C) Distribution of FPA binding sites around PAS that FPA promotes. The upper panel illustrates the distribution of distances between the summits of FPA binding sites and the poly(A) sites promoted by FPA. The RIP‐Seq and PAT‐Seq data for one representative FPA target pre‐mRNA exhibited by the integrative genomics viewer, are also shown in the bottom panel. See also Figure S10 (Supporting Information) for additional examples. D) Schematic diagram of full‐length and truncated FPA proteins is shown in the top panel. Western blot against anti‐FPA and anti‐Tubulin antibodies in WT, *fpa*, and FPA‐GFP transgenic lines is shown in the bottom panel. E) RIP‐Seq metagene profile shows the occupancy of FPA around the poly(A) site region and the dependence on its RRM domains for its binding. Average signal (line) and associated 95% confidence interval (shade) are represented. F) RT‐qPCR analyzes the APA events from three representative pre‐mRNAs in WT, *fpa*, and FPA transgenic lines. The relative RNA abundance in *fpa* or *FPA* transgenic lines was normalized to that in WT. Data is presented as Mean + SD, n = 3 independent biological samples. Two‐tailed Student's *t*‐test was used to analyze the difference between the indicated two samples, ^***^ *p* < 0.001, ^**^ *p* < 0.01, ^*^ *p* < 0.05, not significant (ns).

For pre‐mRNAs whose polyadenylation is mediated by FPA, most binding sites were located in the 3′ UTR and coding regions, with only a small portion in the 5′ UTR (**Figure** **4**A). This binding pattern aligns with the observation that most poly(A) sites are localized in the 3′ UTR. Additionally, most FPA binding sites were generally located downstream of the functional poly(A) site that FPA promotes (Figure 4B).

For FPA‐mediated pre‐mRNAs, most FPA‐binding sites are localized just downstream of the functional poly(A) site of the pre‐mRNA. This feature is clearly illustrated for representative individual pre‐mRNAs (Figure 4C; Figure S10, Supporting Information). Mutations in *FPA* lead to alternative polyadenylation, resulting in increased usage of distal poly(A) sites, decreased usage of proximal poly(A) sites, or both for these representative gene transcripts (Figure 4C; Figure S10, Supporting Information). These findings indicate that FPA binding to these pre‐mRNAs is essential for their correct polyadenylation.

### 2.4 RRM Domains of FPA are Required for FPA to Bind Pre‐mRNA and Regulate Pre‐mRNA Polyadenylation

Given that FPA contains RRM (RNA‐recognition motif) domains, it is important to determine whether these domains are essential for its binding to pre‐mRNA. To address this, we transformed GFP‐tagged truncated FPA lacking the three RRM domains (FPA_ΔRRM_) into the *fpa* mutant background (Figure 4D). There is no obvious difference in subcellular localization or abundance between the full‐length (FPA‐GFP) and truncated version (FPA_ΔRRM_‐GFP) of FPA (Figure S4D, Supporting Information). Genome‐wide analysis revealed that no binding sites were detected for FPA_ΔRRM_. Moreover, the binding sites of full‐length FPA, which are typically located downstream of the functional poly(A) site it promotes, showed no significant binding for FPA_ΔRRM_ (Figure 4E). This indicates that FPA's binding to pre‐mRNAs is dependent on its RRM domains. Additionally, FPA‐mediated polyadenylation of these representative pre‐mRNAs was also found to be dependent on the RRM domains (Figure 4F). These results demonstrate that the RRM domains of FPA are crucial for its binding to pre‐mRNAs and for regulating pre‐mRNA polyadenylation in *Arabidopsis*.

### 2.5 FPA Recognizes GA‐Rich Element in the Pre‐mRNA to Specify the Pre‐mRNA Species via RRM Domains

**Figure 5:**
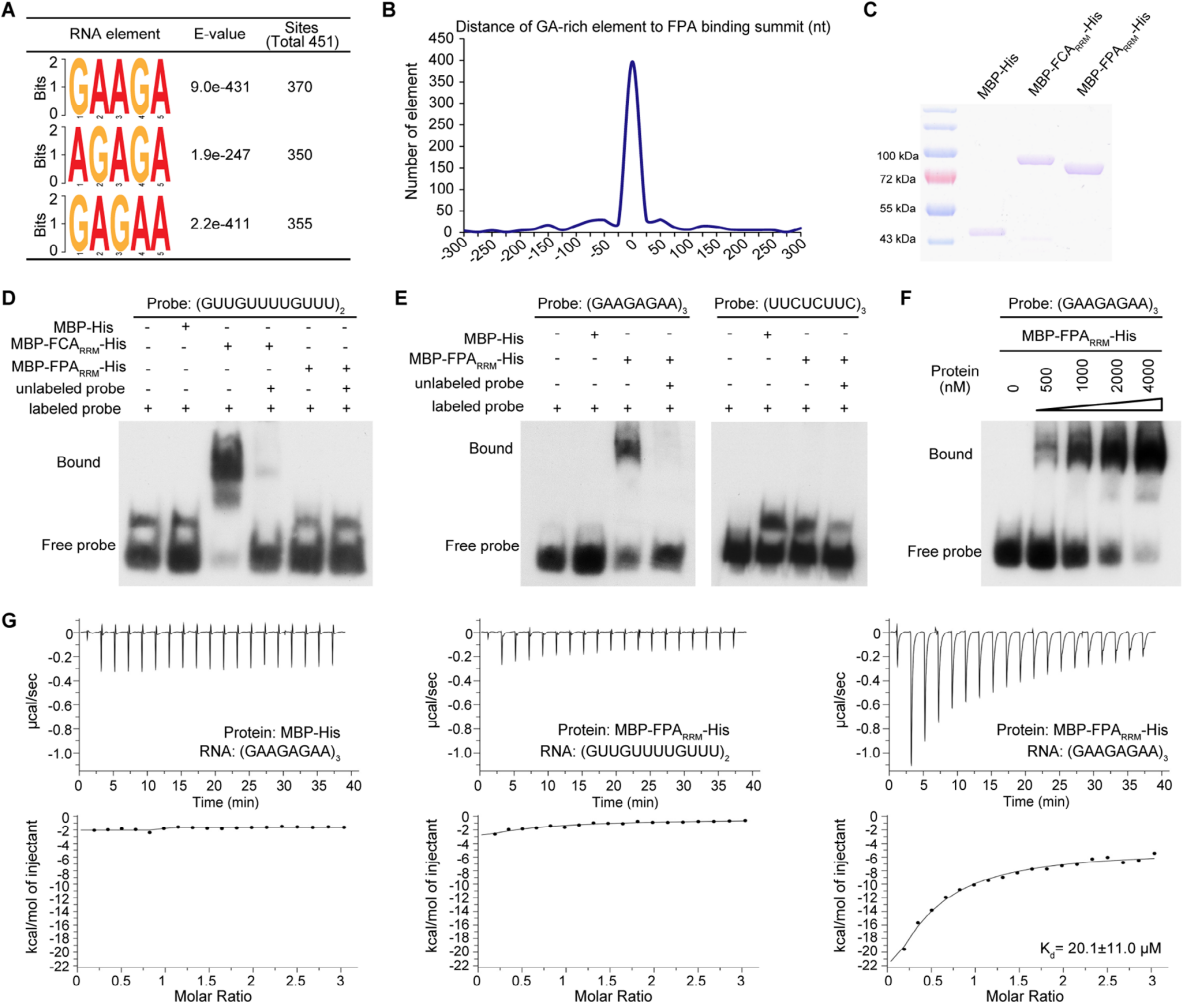
FPA recognizes GA‐rich element in the pre‐mRNA to specify the pre‐mRNA species via RRM domain A) FPA‐binding elements identified by MEME based on RRM domains‐dependent FPA binding sites in FPA‐mediated pre‐mRNAs. B) GA‐rich element localizes in the center of FPA‐binding sites in the pre‐mRNAs. The distribution of distances between the GA‐rich element and the summits of FPA binding sites is shown. C) Purifications of FCA_RRM_ and FPA_RRM_ fusion proteins expressed in *E. coli*. The RRM domains of FCA and FPA, which were fused with MBP and His, were purified. The purified MBP‐His fusion protein was used as a control. D) EMSA assay demonstrates the binding of FCA_RRM_ but not FPA_RRM_ to GU‐rich RNA. Two nm labeled RNA probe and 600 nm purified protein were used, MBP‐His served as the negative control. E) EMSA assay demonstrates the specific binding of FPA_RRM_ to GA‐rich RNA but not to UC‐rich RNA. Two nm labeled RNA probe and 2000 nm purified protein were used, MBP‐His served as the negative control. F) EMSA assay demonstrates the binding of FPA_RRM_ to GA‐rich RNA. Four nm labeled RNA probe and the indicated concentrations of FPA_RRM_ protein were used, the first lane contains 1000 nm MBP‐His as the negative control. G) ITC assay demonstrates the binding of FPA_RRM_ to GA‐rich RNA but not GU‐rich RNA. MBP‐His was used as the negative control.

To understand how the specificity of FPA's binding to pre‐mRNAs is determined, we sought to identify a consensus sequence for FPA binding within the binding sites of FPA‐mediated pre‐mRNAs. Given that the RRM domains are essential for FPA's binding to pre‐mRNAs (Figure 4), we analyzed the 427 pre‐mRNAs that exhibited DEPAS between WT and *fpa*, focusing on the 536 FPA‐binding sites. Using FPA_ΔRRM_‐GFP/*fpa* as a control, we identified 451 binding sites where the FPA full‐length protein showed more than two‐fold enrichment compared to FPA_ΔRRM_. This indicated that FPA's binding to these sites is dependent on its RRM domains. We observed that a GA‐rich element was present in most of these RRM‐dependent FPA‐binding sites (**Figure** **5**A), and this consensus cis‐element is centrally located within the FPA‐binding site sequence in the pre‐mRNA (Figure 5B).

To verify FPA's binding to the GA‐rich element, we expressed a truncated FPA containing the three RRM domains in *E. coli* (Figure 5C). We then tested its binding properties using an EMSA assay. As FCA was previously reported to bind a GU‐rich element,^[^ ^51^, ^52^ ^]^ we used FCA as a positive control. FCA is indeed bound to the GU‐rich element, and this binding was competed by an unlabeled GU‐rich element. In contrast, FPA did not bind to the GU‐rich element (Figure 5D). However, FPA is clearly bound to the GA‐rich element, and this binding was competed by an unlabeled GA‐rich element. The binding signal increased with the amount of FPA protein, but FPA did not bind to the antisense of the GA‐rich element (UC‐rich element) (Figure 5E,F). These results suggest that FPA's binding to the GA‐rich element is specific, and different RNA‐binding proteins bind to distinct pre‐RNAs with differences in their consensus elements.

To further confirm FPA's binding to the GA‐rich element, we performed an ITC assay. We observed that MBP (maltose‐binding protein) did not bind to the GA‐rich element, while FPA did not bind to the GU‐rich element but bound to the GA‐rich element with a dissociation constant (K_d_) of ≈20 µm (Figure 5G). Collectively, these results indicate that FPA specifically binds to the GA‐rich element in vitro.

### 2.6 CFII Complex Binds to GA‐Rich Element‐Containing Pre‐mRNAs to Regulate their Alternative Polyadenylation in an FPA‐Dependent Manner in *Planta*

We next investigated whether the specific binding of FPA to the GA‐rich element occurs in *planta* and whether recognition of this element is required for the CFII complex to mediate alternative polyadenylation of pre‐mRNA. To this end, we selected a set of representative pre‐mRNAs from the *Arabidopsis* genome for detailed analysis.

**Figure 6:**
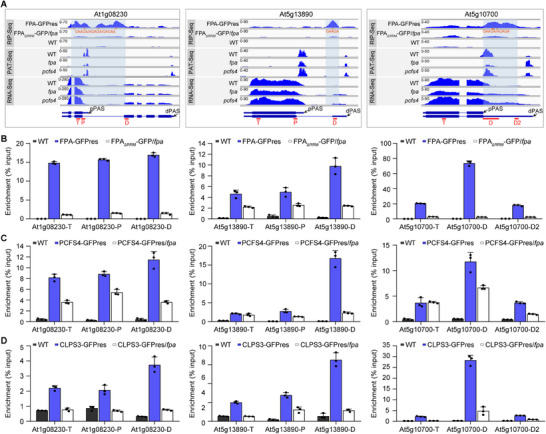
CFII complex promotes proximal poly(A) site usage by recognizing the GA‐rich element in pre‐mRNAs in an FPA‐dependent manner A) FPA binds to the GA‐rich element‐containing region downstream of proximal PAS and is required for regulation of APA for representative pre‐mRNAs in Figure 3 based on the RIP‐Seq, PAT‐Seq, and RNA‐Seq data exhibited by Integrative Genomics Viewer. The red lines under the structure of pre‐mRNA represent the position amplified by mRNA by qPCR, the orange dots indicate the position of the GA‐rich element. B–D) RIP‐RT‐qPCR analysis demonstrates the binding of FPA (B), PCFS4 (C), and CLPS3 (D) to the three representative target pre‐mRNAs, and the dependence of the RRM domain for FPA (B), or dependence of FPA for PCSF4 (C) and CLPS3 (D) for their binding to the targets. The IPs were normalized to the input. Three biological replicates were done with similar results, one representative result is shown. Data is presented as Mean + SD, n = 3 technical replicates. Primer sequences are provided in Table S5 (Supporting Information). Also see Figures S10 and S11 (Supporting Information).

First, we examined their RIP‐Seq and PAT‐Seq results using IGV (Integrative Genomics Viewer) images. We observed that FPA binds to regions downstream of the proximal poly(A) site, and these FPA‐binding sites indeed contain the GA‐rich element. Additionally, mutations in *FPA* or *PCFS4* lead to a shift in poly(A) site usage from proximal to distal sites (**Figure** **6**A; Figure S11A, Supporting Information).

Next, we confirmed these observations using RIP‐RT‐qPCR assays for individual representative pre‐mRNAs in *planta*, and whether the RRM domains of FPA are required for FPA to bind to those pre‐mRNAs (Figure 6B; Figure S11B, Supporting Information). Using PCFS4‐GFP and CLPS3‐GFP transgenic lines (Figure S4A,E, Supporting Information), we further demonstrated that both PCFS4 and CLPS3, components of the CFII complex, exhibit a similar binding footprint to FPA for these representative pre‐mRNAs. Their binding to these pre‐mRNAs is dependent on FPA (Figure 6C,D; Figure S11C,D, Supporting Information). This indicates that FPA is essential for the CFII complex to bind to pre‐mRNAs and facilitate cleavage and polyadenylation. Collectively, these results demonstrate that the in vitro findings from Figure 5 are consistent with in vivo observations. FPA collaborates with PCFS4 and CLPS3 to recognize GA‐rich signals, thereby promoting proximal poly(A) site usage.

FPA binding sites are generally enriched with GA‐rich motifs. Among the 3840 FPA binding sites identified, 3650 (≈95%) contain a GA‐rich element. Genome‐wide analysis results demonstrate that when FPA binding is detected downstream of a PAS, the usage of these PAS changes more significantly in *fpa* compared to the wild type (Figure S12A, Supporting Information). This indicates that FPA binding downstream of PAS has a pronounced promoting effect on PAS usage. Additionally, when FPA binding sites are located within 40 nucleotides (nt) upstream of a PAS, particularly between 20 and 40 nt, the promoting effect on upstream PAS usage is more evident (Figure S12B, Supporting Information). Moreover, when a GA‐rich element is present downstream of a PAS, the usage of that PAS changes more significantly in the *fpa* compared to the wild type (Figure S12C, Supporting Information). This suggests that the presence of a downstream GA‐rich element also has a pronounced promoting effect on PAS usage. Furthermore, when the GA‐rich element is within 60 nt upstream of a PAS, especially between 20 and 40 nt, the promoting effect on PAS usage is even more pronounced (Figure S12D, Supporting Information). Overall, FPA binding promotes the use of upstream PAS, with the strongest enhancement observed when the PAS is positioned 20–40 nt upstream of the FPA binding site (Figure S12E, Supporting Information). These results indicate that both FPA binding and the presence of GA‐rich elements downstream of PAS promote the usage of these PAS. Moreover, the combined presence of a GA‐rich motif within 20–40 nt downstream of a PAS, along with FPA binding, has an even stronger promoting effect.

**Figure 7:**
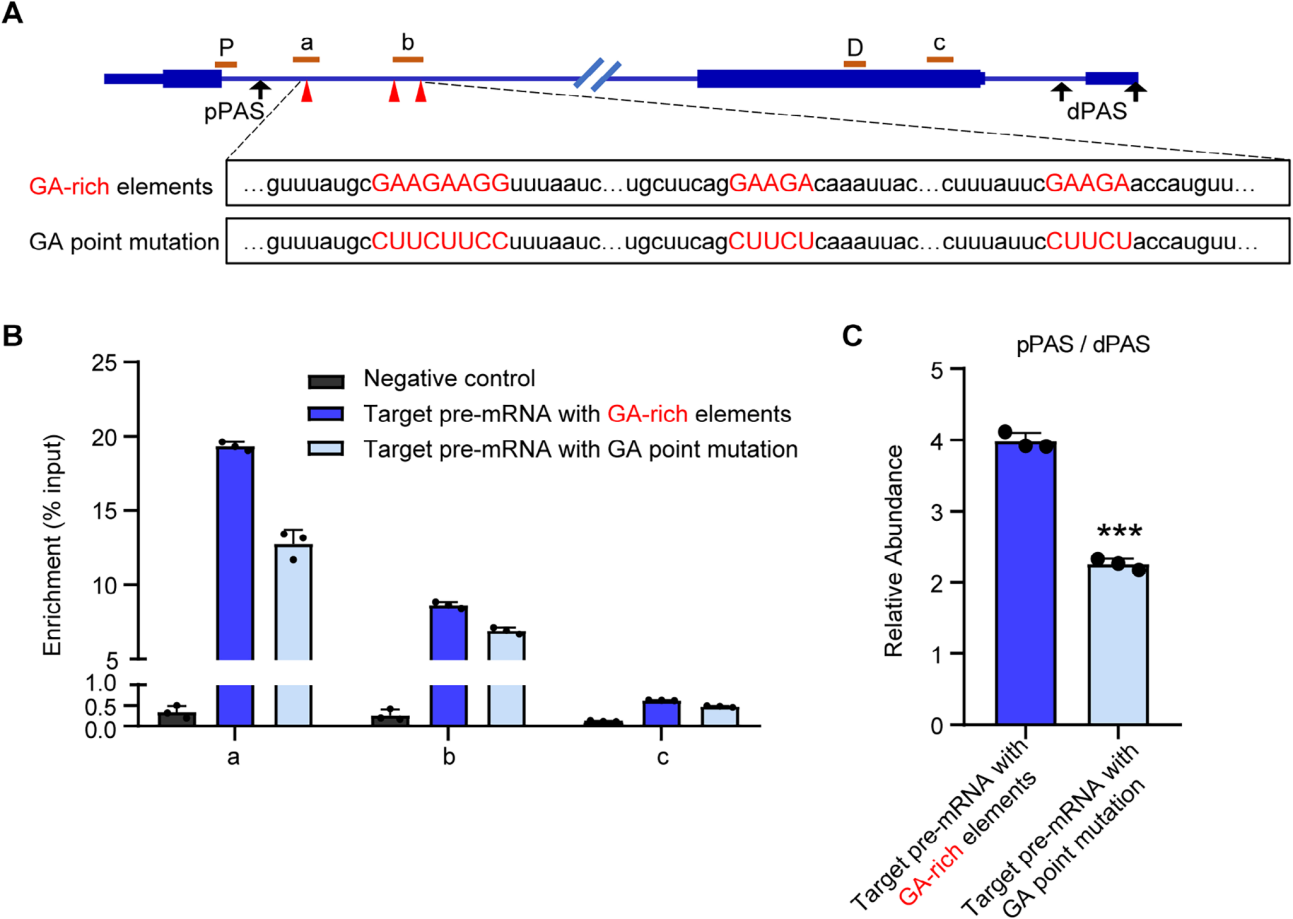
GA‐rich element is necessary for FPA binding and APA regulation in vivo. A) Diagram of the FPA‐targeted pre‐mRNA with GA‐rich elements or GA point mutations. The brown lines above the pre‐mRNA structure represent the positions of amplified mRNA by qPCR. Red triangles indicate the positions of three GA‐rich elements, with their sequences displayed below the pre‐mRNA schematic. Black arrows represent poly(A) sites in the pre‐mRNA: pPAS (proximal poly(A) site) and dPAS (distal poly(A) site). Primer sequences are provided in Table S5 (Supporting Information). B) RIP‐RT‐qPCR analysis demonstrates FPA binding to the GA‐rich element or the GA point mutation pre‐mRNA. The immunoprecipitated RNAs by FPA‐GFP were normalized to the input, with wild type plants used as the negative control. Three independent biological replicates were performed with similar results; one representative result is shown. Data are presented as Mean + SD, n = 3 technical replicates. For each of the two transgenic events of target pre‐mRNA, six independent transgenic lines were pooled for RIP‐RT‐qPCR analysis. C) RT‐qPCR analyses of the relative pPAS and dPAS isoforms of the FPA‐targeted pre‐mRNA. Data are presented as Mean + SD, n = 3 biologically independent samples. Two‐tailed Student's *t*‐test, ^***^ *p* < 0.001. P and D were used to detect the pPAS and dPAS isoforms, respectively. For each of the two transgenic events of target pre‐mRNA, the relative levels of pPAS and dPAS isoforms were presented as the mean values derived from six independent lines.

To further elucidate the promoting effect of FPA‐specific binding to GA‐rich elements on the usage of upstream PAS in *Arabidopsis*, we investigated the impact of GA‐rich element mutations on FPA binding and the usage of upstream PAS. We selected a target pre‐mRNA of FPA that contains three GA‐rich elements downstream of its proximal poly(A) site. We introduced both the wild‐type sequence containing the GA‐rich elements and a point‐mutated sequence, in which GA was replaced with CT, respectively, into *Arabidopsis* (**Figure** **7**A). We found that FPA binds to the region downstream of the proximal PAS containing the GA‐rich elements. However, when the GA‐rich elements were mutated to CT, FPA binding to this region was significantly reduced (Figure 7B), and the usage rate of the proximal PAS also decreased significantly (Figure 7C). These results further demonstrate that in *Arabidopsis*, the presence of GA‐rich elements and FPA binding to these elements significantly promotes the usage of upstream PAS.

### 2.7 CFII Complex Recognizes the Fourth Consensus Element Downstream the Functional Poly(A) Site for Alternative Polyadenylation of Pre‐mRNA in *Arabidopsis*

Both FPA‐ and PCFS4‐mediated genome‐wide alternative polyadenylation suggest that FPA recognizes a GA‐rich element in pre‐mRNA to specify poly(A) sites (Figures 5, 6, and 7; Figure S11A,B, Supporting Information). It is known that the cleavage and polyadenylation machinery sorts pre‐mRNAs and correctly cleaves and forms poly(A) tails by recognizing conserved elements around the functional poly(A) site in both vertebrates and plants.^[^ ^26^ ^]^ In vertebrates, the CPSF complex recognizes the core hexamer AAUAAA element (or variants) in pre‐mRNA, the CFI complex binds to the UGUA element upstream, and the CstF complex binds to the U‐rich or (G+U)‐rich region downstream of the poly(A) site.^[^ ^26^ ^]^ In plants, the near upstream element (NUE), far‐upstream element (FUE), and cleavage element (CE) are equivalent to AAUAAA, UGUA, and U‐ (or G+U)‐rich elements or their variants, respectively.^[^ ^28^, ^29^, ^30^, ^31^, ^32^ ^]^

**Figure 8:**
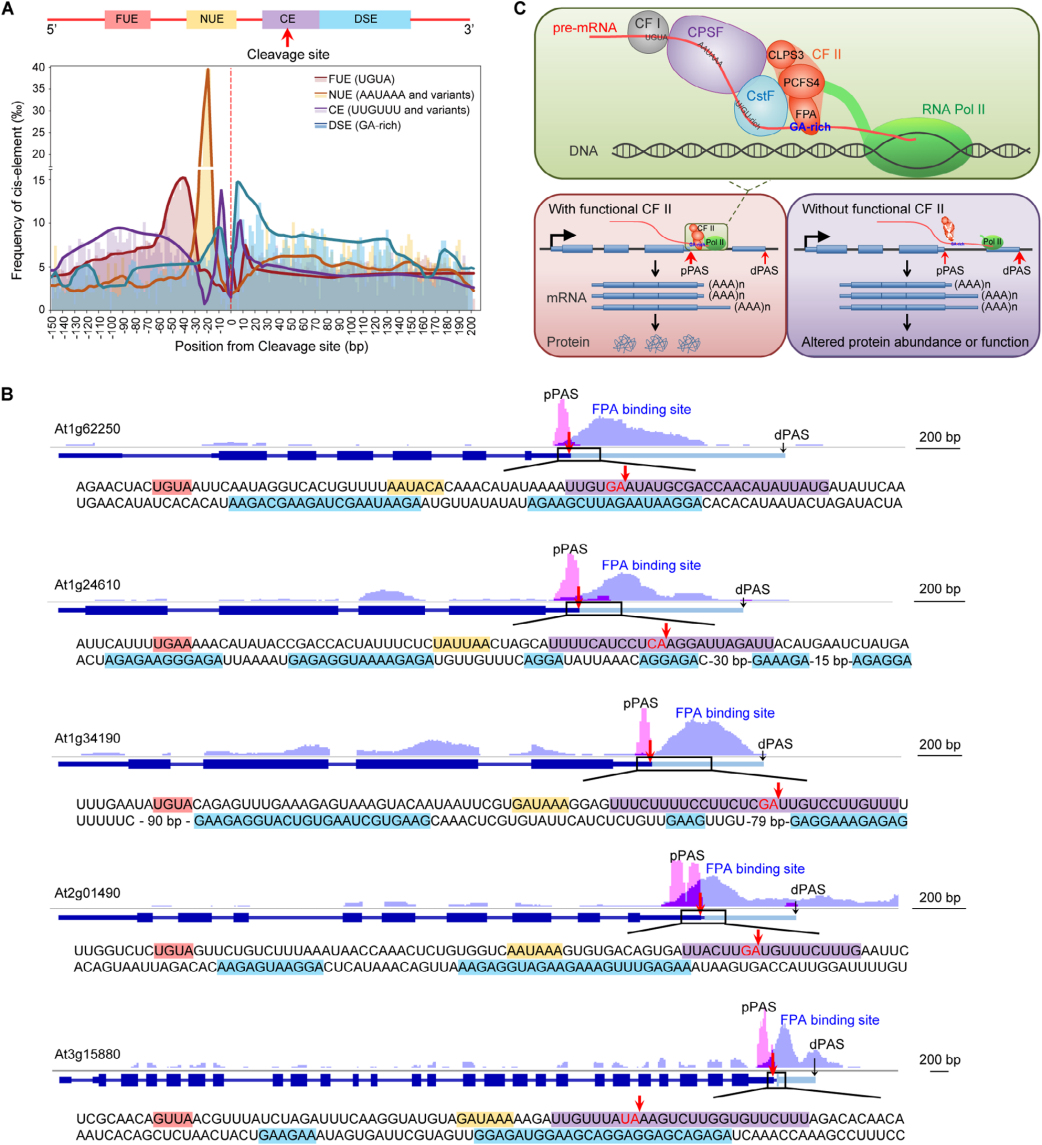
CFII complex recognizes the fourth consensus element downstream the functional poly(A) site of pre‐mRNA in *Arabidopsis* A) The conserved elements of the three known poly(A) signals and the GA‐rich element that FPA binds downstream of the functional PAS (indicated as 0) for FPA‐mediated pre‐mRNAs. Density plots show the distributions of the four poly(A) signal elements centered around FPA‐promoted PAS, ranging from −150 to +200 nt. B) The four poly(A) signals around the proximal PAS are shown from FPA‐mediated representative pre‐mRNAs. Poly(A) sites from PAT‐Seq data and FPA binding sites from RIP‐Seq data, which are above the gene structure, are exhibited by Integrative Genomics Viewer. The pre‐mRNA sequences around the proximal poly(A) site are shown under the gene structure. The sequence with red background: FUE (mainly UGUA); the sequence with yellow background: NUE (AAUAAA or close variants); the sequence with purple background: CE (UUGUUU or close variants); the sequence with blue background: DSE (GA‐rich element that FPA binds). Red font and arrow: cleavage site. pPAS: proximal poly(A) site, dPAS: distal poly(A) site. C) A working model for FPA‐CFII‐mediated APA. FPA specifically binds to the GA‐rich element region downstream of the proximal poly(A) site of target pre‐mRNAs. FPA physically interacts with the CFII complex and bridges the binding of PCFS4 and CLPS3 to target pre‐mRNAs, working collectively with other complexes to define functional poly(A) sites and fulfill cleavage and polyadenylation. The CFII complex promotes proximal poly(A) site usage and prevents 3′ extension, thereby regulating the protein abundance or function of target RNAs.

Analysis of the conserved elements in pre‐mRNAs whose alternative polyadenylation is regulated by FPA and PCFS4 revealed that these three consensus elements are also present in *Arabidopsis* pre‐mRNAs, with similar locations around the functional poly(A) site as reported in animals and plants (**Figure** **8**A). However, a fourth consensus GA‐rich element is recognized by the FPA‐containing CFII complex and is located downstream of the poly(A) site (downstream element, DSE) in the pre‐mRNA (Figure 8A). This observation is further illustrated by FPA‐regulated representative pre‐mRNAs, in which the FPA binding site contains the GA‐rich element, and these FPA‐bound GA‐rich elements are located downstream of the proximal poly(A) site in the pre‐mRNA (Figure 8B).

Therefore, the CFII complex, similar to the other three complexes of the cleavage and polyadenylation machinery, recognizes a consensus element around the functional poly(A) site to fulfill its role in alternative polyadenylation of pre‐mRNA. The consensus element recognized by the CFII complex is located downstream of the poly(A) site it promotes in pre‐mRNA (Figure 8C).

## 3 Discussion

Pre‐mRNA cleavage and polyadenylation is a critical step in the generation of mature mRNA, particularly for pre‐mRNAs with multiple poly(A) sites. Selecting the correct poly(A) site is essential for regulating gene expression. Alternative polyadenylation has been shown to influence mRNA metabolism, protein diversification, and protein localization, thereby affecting biological function.^[^ ^1^, ^2^, ^3^, ^4^ ^]^ Additionally, alternative polyadenylation is highly cell type‐ and tissue‐specific and is regulated by environmental cues.^[^ ^5^, ^6^, ^11^, ^12^, ^13^, ^14^, ^15^, ^16^ ^]^ While the significance of alternative polyadenylation is well recognized, the molecular mechanisms underlying the selection of alternative poly(A) sites are not fully understood.

Here, we provide novel insights into the identification and recognition of the fourth and downstream cis‐element of the poly(A) site. We demonstrate that the RNA‐binding activity of FPA, in conjunction with the CFII complex, leads to the selection of functional alternative polyadenylation sites in *Arabidopsis*. This study elucidates a key aspect of the molecular regulation of alternative polyadenylation.

### 3.1 CFII Complex Physically Interacts and Closely Associates with an RNA‐Binding Protein FPA in *Arabidopsis*

The specificity and sites of pre‐mRNA polyadenylation are primarily defined by conserved cis‐elements in pre‐mRNA. The CPSF complex recognizes the core hexameric consensus element AAUAAA (or close variants) through direct interaction between the RNA‐binding protein WDR33/CPSF30 and AAUAAA.^[^ ^22^, ^23^, ^33^, ^34^ ^]^ The UGUA element upstream of AAUAAA is bound by CFI25, an RNA‐binding protein and a component of the CFI complex.^[^ ^53^ ^]^ Meanwhile, the U‐rich and GU‐rich auxiliary elements downstream of AAUAAA are bound by the RNA‐binding protein CstF64 or τCSTF64, components of the CstF complex in vertebrates.^[^ ^54^, ^55^ ^]^ These studies indicate that each complex recognizes a specific consensus element around the functional poly(A) site through interactions involving RNA‐binding proteins. However, the core polyadenylation signal AAUAAA (or close variants) occurs every 500 nucleotides on average in pre‐mRNAs but is not used for cleavage and polyadenylation at these sites.^[^ ^4^, ^11^ ^]^ This suggests that additional elements are necessary to collectively define a functional poly(A) site.

Among the four connected complexes in the cleavage and polyadenylation machinery, the CFII complex is poorly characterized. The CFII complex is composed of Pcf11 and Clp1 in mammals and their homologs in plants (Figure 1A,B).^[^ ^4^, ^45^, ^46^ ^]^ Although the CFII complex is required for alternative polyadenylation, no subunit with RNA‐binding activity has been identified within it, and no specific sequences around the poly(A) site have been shown to interact with it in eukaryotes.^[^ ^4^, ^31^, ^32^, ^34^ ^]^ Our work confirms that PCFS4 and CLPS3, the homologs of human Pcf11 and Clp1, respectively, form a functional CFII complex in *Arabidopsis* (Figure 1A,B; Tables S1 and S2, Supporting Information). They directly and physically interact both in vitro and in vivo (Figure S3A–C). Similar to Pcf11 in human cells,^[^ ^50^ ^]^ PCFS4 enhances transcription termination and increases the usage of proximal poly(A) sites, resulting in shorter mRNA isoforms (Figures 2 and 3; Figure S8, Supporting Information). This pattern differs from that regulated by the CFI complex.^[^ ^56^, ^57^, ^58^ ^]^ These results indicate that PCFS4 and CLPS3 form a functional CFII complex in *Arabidopsis*. Additionally, while a mutation in *CLPS3* is embryonic lethal, a mutation in *PCFS4* is viable,^[^ ^45^, ^46^ ^]^ suggesting that other homologs of PCFS4, such as PCFS1, PCFS2, or PCFS5, may also interact with CLPS3 to form functional CFII complexes in *Arabidopsis*.

Furthermore, Parker et al.^[^ ^41^ ^]^ observed that FPA co‐purified with several polyadenylation factors from CPSF, CstF, and CFII complexes, as well as other factors involved in the autonomous pathway for floral transition. Our pair‐wise co‐purification analysis indicates that both the CFII complex and FPA are closely associated and included in the same complex (Figure 1A,B; Tables S1 and S2, Supporting Information). Although we cannot exclude the possibility that FPA may exhibit weak interactions with other cleavage and polyadenylation subcomplexes, our data confirm that it is the CFII complex, rather than CPSF or CstF, that physically interacts with FPA. This interaction is mediated by a direct association between PCFS4 and FPA, with PCFS4 bridging the interaction between FPA and CLPS3 in *Arabidopsis* (Figure 1; Figures S1 and S3, Supporting Information). Thus, FPA and the CFII complex are in the same complex, with FPA acting as a closely associated factor and accessory protein for the CFII complex to fulfill its function in alternative polyadenylation. Therefore, the CFII complex physically interacts with an RNA‐binding protein in *Arabidopsis*.

### 3.2 FPA Mediates Alternative Polyadenylation by Interaction with CFII Complex

In this study, we observed that FPA regulates alternative polyadenylation for a subset of pre‐mRNAs in the *Arabidopsis* genome (Figure 2). Previous reports have shown that FPA regulates alternative polyadenylation not only for its own pre‐mRNA but also for several other pre‐mRNAs.^[^ ^38^, ^41^, ^43^, ^44^ ^]^ Additionally, mutations in *FPA* lead to defects in alternative polyadenylation for a set of pre‐mRNAs in the *Arabidopsis* genome,^[^ ^40^, ^42^ ^]^ indicating that FPA is a cleavage and polyadenylation factor in *Arabidopsis*. Although FY, the homolog of human WDR33 in *Arabidopsis*, and FCA are also floral transition regulators in the autonomous pathway and are components of the CPSF complex that interact to regulate alternative polyadenylation for some pre‐mRNAs,^[^ ^59^, ^60^ ^]^ FPA and FCA/FY control poly(A) site selection independently of each other.^[^ ^39^ ^]^ Therefore, the molecular mechanism by which FPA achieves its function in pre‐mRNA alternative polyadenylation was not clear.

In the present study, we confirmed that FPA selectively binds to pre‐mRNAs containing a GA‐rich element through recognition of the GA‐rich element both in vitro and in vivo. The RRM domains in FPA are responsible for this binding (Figures 4, 5, 6, 7, 8; Figures S10 and S11, Supporting Information). Additionally, FPA physically interacts with the CFII complex by directly interacting with PCFS4 (Figure 1), and the SPOC domain in FPA is responsible for this direct interaction (Figure 1; Figure S1, Supporting Information). Whereas mutations in distinct 3′ cleavage and polyadenylation factors lead to diverse alternative polyadenylation (APA) defects (Figure S13, Supporting Information), the *fpa* and *pcfs4* exhibit similar APA defects. Moreover, the DEPAS regulated by FPA and PCFS4 display similar regulatory patterns (Figures 2 and 3, Supporting Information). Furthermore, FPA mediates the recognition between the CFII complex and the fourth cis‐element downstream of the poly(A) site in pre‐mRNA, which is required for correct polyadenylation (Figures 6, 7, 8; Figure S11, Supporting Information).

Thus, our work reveals the molecular mechanism for FPA‐regulated alternative polyadenylation: FPA interacts with the CFII complex and recruits it to pre‐mRNAs containing a specific consensus element, thereby producing a mature mRNA with an appropriate poly(A) tail (Figure 8C).

### 3.3 Novel Poly(A) Signal Recognized by CFII and FPA is Located Downstream of Ploy(A) Site in Pre‐mRNA

Previous studies have identified three poly(A) signals in pre‐mRNA: AAUAAA, UGUA, and U‐rich (or GU‐rich) elements in animals, and their respective variants‐near upstream element (NUE), far‐upstream element (FUE), and cleavage element (CE)‐in plants.^[^ ^28^, ^29^, ^30^, ^31^, ^32^ ^]^ These three cis‐elements are located upstream of or at the poly(A) site. However, no downstream cis‐element of the poly(A) site has been identified in plants so far, despite its necessity for correct poly(A) site selection in pre‐mRNA. Here, we identified that FPA binds downstream of proximal polyadenylation sites in *Arabidopsis*. Additionally, FPA binding sites are enriched for GA‐rich elements, with 95% of the 3840 identified FPA binding sites containing at least one of the three GA‐rich elements characterized in Figure 5A. We demonstrate that FPA specifically recognizes GA‐rich elements downstream of poly(A) sites, in addition to the other three known conserved elements in FPA‐mediated pre‐mRNAs (Figure 8A). For the four consensus elements we identified in *Arabidopsis*, consistent with observations in mammals and plants: The core hexameric CE (AAUAAA or close variants) is located ≈20 nt upstream of the functional poly(A) site. FUE (UGUA element) is positioned ≈20–30 nt upstream of the hexamer and 40–50 nt upstream of the functional poly(A) site. NUE (U‐rich element or variants) is ≈10 nt downstream of the hexamer and 10 nt upstream of the functional poly(A) site. The fourth consensus element, the GA‐rich element recognized by the FPA‐containing CFII complex, is ≈30–50 nt downstream of the hexamer and 10–30 nt downstream of the functional poly(A) site (Figure 8).

Thus, the GA‐rich element recognized by the FPA‐containing CFII complex is the first identified downstream element (DSE) and a novel poly(A) signal downstream of the poly(A) site in pre‐mRNA. Based on our observations in *Arabidopsis*, a new geometric model for plants is emerging, where the CPSF complex is downstream of the CFI complex but upstream of the CstF complex, while the CFII complex with FPA is downstream of the CstF complex along the pre‐mRNA (Figure 8C).

We also observed that FPA regulates alternative polyadenylation for a subset of pre‐mRNAs, fewer than those regulated by PCFS4 in the genome (Figures 2 and 3, Supporting Information). There are some differences in the pre‐mRNA species regulated by PCFS4 and FPA (Figures 2 and 3, Supporting Information), implying that FPA is not the only RNA‐binding protein that works with the CFII complex for alternative polyadenylation. Potentially, other RNA‐binding proteins may interact with the CFII complex similarly to FPA for other sets of pre‐mRNAs with different downstream elements from the fourth consensus identified in FPA‐mediated pre‐mRNAs. Unlike the other three polyadenylation complexes, which recognize consensus elements through constant RNA‐binding proteins, the CFII complex recognizes the consensus element through an alternative RNA‐binding protein, such as FPA.

The fourth element and the downstream element were not clearly identified previously for several reasons. One reason is that the fourth element (the downstream element) is included in a subset of pre‐mRNAs in the genome and may have been diluted in whole‐transcriptome analyses. For example, the GA‐rich element identified in this work is specific to FPA‐mediated pre‐mRNAs and may have been diluted and not ranked highly in a transcriptome‐wide compilation of poly(A) signals in *Arabidopsis*.^[^ ^29^, ^61^ ^]^

Although our results are based on evidence from the plant system, FPA is a conserved Spen family RNA‐binding protein, and Spen family RNA‐binding proteins are present in eukaryotes from plants to humans and yeast,^[^ ^62^, ^63^ ^]^ such as SHARP, RBM15, and RBM15B in humans.^[^ ^63^ ^]^ Both the molecular domain architecture and the structure of the SPOC domain of Spen family proteins are conserved across eukaryotes (Figure S2, Supporting Information). Therefore, Spen family RNA‐binding proteins from other species are potential candidates for RNA‐binding proteins that act as alternative components or accessory proteins of the CFII complex. Further studies in other systems will likely resolve this issue in the near future.

## 4 Conclusion

Alternative polyadenylation generates mRNA isoforms with alternative 3′ ends and is recognized as a major mechanism of gene expression regulation. The specificity and site selection of polyadenylation are primarily determined by highly conserved cis‐elements in pre‐mRNA, which are collectively recognized by four polyadenylation complexes. In this study, we identified a cis‐element downstream of the poly(A) site in pre‐mRNA that is recognized by the polyadenylation CFII complex. This identification was achieved through the RNA‐binding activity of FPA. We confirmed that an RNA‐binding protein, such as FPA, acts as an accessory protein for the CFII complex, facilitating its recognition of the downstream cis‐element in pre‐mRNAs during alternative polyadenylation. This work not only reveals the molecular mechanism underlying FPA‐mediated alternative polyadenylation in *Arabidopsis* but also identifies a novel downstream cis‐element and its recognition mechanism by the CFII complex for alternative polyadenylation of pre‐mRNAs in eukaryotes.

## 5 Experimental Section

### Plant Materials

The *Arabidopsis thaliana* accession Col‐0 was used as the wild‐type (WT). The *fpa* mutant (CS854907) was obtained from the ABRC, while the *pcfs4* (SALK_102 934) and *clps3* (SALK_02 5156) mutants were described previously.^[^ ^45^, ^46^ ^]^ All mutations were confirmed by PCR.

For FPA‐GFPres plants (Figure S4C, Supporting Information), a construct containing the native promoter‐driven *FPA* genomic DNA (3700 bp *FPA* native promoter + 4590 bp *FPA* coding region + *GFP* CDS + 1355 bp 3′ end downstream of *FPA* stop codon + nos terminator) was generated and cloned into the pCAMBIA2300 vector. This construct was then transformed into the *fpa* mutant background.

For FPA_ΔRRM_‐GFP/*fpa* Plants, a truncated *FPA* genomic coding region (2080 bp, with a 2510 bp deletion from the start codon) replaced the full‐length *FPA* genomic coding region in the *FPA‐GFP* construct. This modified construct was transformed into the *fpa* mutant background.

For FPA‐MYCres Plants (Used in IP‐MS), a construct containing the native promoter‐driven *FPA* genomic DNA (2500 bp native promoter + 4590 bp *FPA* coding region + 9× *MYC* CDS + 1355 bp 3′ end downstream of *FPA* stop codon + nos terminator) was generated and cloned into the pCAMBIA2300 vector. This construct was then transformed into the *fpa* mutant background.

For PCFS4‐MYCres Plants (Figure S4E, Supporting Information), a construct containing the native promoter‐driven *PCFS4* (1449 bp *PCFS4* native promoter + 2427 bp *PCFS4* CDS + 12× *MYC* CDS + nos terminator) was generated and cloned into the pCAMBIA1300 vector. This construct was then transformed into the *pcfs4* mutant background.

For PCFS4‐GFPres Plants (Figure S4E, Supporting Information), a construct containing the native promoter‐driven *PCFS4* genomic DNA (3000 bp *PCFS4* native promoter + 3332 bp *PCFS4* coding region + *GFP* CDS + 1000 bp 3′ end downstream of *PCFS4* stop codon + nos terminator) was generated and cloned into the pCAMBIA1300 vector. This construct was then transformed into the *pcfs4* mutant background. Two representative PCFS4‐GFPres lines were crossed with *fpa* to generate the PCFS4‐GFPres/*fpa* plant.

For CLPS3‐FLAGres Plants (Figure S4A, Supporting Information), a construct containing the native promoter‐driven *CLPS3* genomic DNA (2000 bp *CLPS3* native promoter + 2360 bp coding region + 3× *FLAG* CDS + 1000 bp 3′ end downstream of *CLPS3* stop codon) was generated and cloned into the pCAMBIA1300 vector. This construct was then transformed into the *clps3* mutant background.

For CLPS3‐GFPres Plants (Figure S4A, Supporting Information), a construct containing the native promoter‐driven *CLPS3* genomic DNA (3000 bp *CLPS3* native promoter + 2360 bp *CLPS3* coding region + *GFP* CDS + 1000 bp 3′ end downstream of *CLPS3* stop codon) was generated and cloned into the pCAMBIA1300 vector. This construct was then transformed into the *clps3* heterozygous background (*clps3* (±)). Two representative CLPS3‐GFPres lines were crossed with *fpa* to generate the CLPS3‐GFPres/*fpa* plant.

Single‐copy insertion and homozygous transgenic lines were characterized by antibiotic resistance: kanamycin resistance for *FPA* transgenic lines and hygromycin resistance for *PCFS4* and *CLPS3* transgenic lines.

### Growth Conditions

*Arabidopsis thaliana* seeds were sterilized and subjected to a 4 °C stratification for 2 days. They were then plated on Murashige & Skoog medium (MS medium, M5519, Sigma) supplemented with 0.3% Phytagel (P8169, Sigma) and 1% sucrose. The plates were placed in a growth chamber (CU36L5, Percival Scientific) under long‐day conditions (16 h light [22 °C]/8 h dark [20 °C] with a light intensity of 130 µmol m^−2^ s^−1^). *Arabidopsis* adult plants and *Nicotiana benthamiana* (tobacco) plants were grown in a greenhouse under a 16 h light (22 °C)/8 h dark (20 °C) photoperiod with a light intensity of 120 µmol m^−2^ s^−1^.

### RNA Isolation and PAT‐Seq

Total RNA was isolated from 9‐day‐old WT, *fpa*, and *pcfs4* plants (grown on 1/2 MS medium under long‐day conditions) using the RNeasy Plant Mini Kit (74 904, Qiagen). The RNA was treated with RNase‐free DNase (M610A, Promega) at 37 °C for 30 min to remove any potential DNA contamination. Three micrograms of DNA‐free RNA were used for strand‐specific RNA‐Seq (conducted by Novogene).

The PAT‐Seq libraries were constructed with modifications to a previously described method.^[^ ^64^ ^]^ Briefly, 4 µg of DNA‐free total RNA were fragmented in 5× first‐strand buffer (Invitrogen) at 94 °C for 4 min. Fragments with poly(A) tails were enriched using oligo(dT)^25^ beads (NEB). Reverse transcription was performed using barcoded oligo(dT)_18_ adaptors and SuperScript III Reverse Transcriptase (Invitrogen) for 2 h. A 5′ adaptor (with the last nucleotide modified by locked nucleic acid) was added for template switching. The cDNA was purified using AMPURE XP beads (Beckman) and eluted in DEPC‐treated water. Two rounds of PCR with each round of 5 cycles were performed using Phire II (Thermo Fisher Scientific) to generate the final PAT‐Seq libraries. Fragments of 300–500 bp were purified and sequenced on the Illumina HiSeq 2500 platform (SE50, College of the Environment and Ecology, Xiamen University). Three independent biological replicates were conducted for sample preparation, RNA isolation, and sequencing.

### Poly(A) Site Analysis

Poly(A) sites were identified from the PAT‐Seq data as previously described^[^ ^64^, ^65^ ^]^ with modifications. Briefly, raw data were processed using fastp^[^ ^66^ ^]^ (https://github.com/OpenGene/fastp) and PRINSEQ^[^ ^67^ ^]^ (http://prinseq.sourceforge.net). Low‐quality bases were removed, and barcodes and poly(T) sequences at the beginning of the reads were trimmed. Clean reads were mapped to the *Arabidopsis* reference genome (TAIR10, www.arabidopsis.org) using HISAT2^[^ ^68^ ^]^ (http://daehwankimlab.github.io/hisat2/). Only uniquely aligned reads (extracted by samtools^[^ ^69^ ^]^ (http://www.htslib.org)) were used for further analysis. Potential internal priming reads were filtered out based on a previously described protocol.^[^ ^29^ ^]^ Due to intrinsic heterogeneity, mapped poly(A) tags (PATs) within 24 nucleotides were pooled together and defined as a poly(A) site (PAS). To facilitate the assignment of PAS to annotated genes, genes with annotated 3′ UTRs were extended by 120 nucleotides, and genes without annotated 3′ UTRs were extended by 338 nucleotides.^[^ ^65^ ^]^ PAS with total reads among all nine samples < 10, or with < 3 reads in any single sample of WT, *fpa*, or *pcfs4*, were discarded. Remaining PAS were compared to poly(A) sites identified in published direct RNA sequencing (DRS) data^[^ ^38^ ^]^ and nanopore direct RNA sequencing (ONT DRS) data^[^ ^70^ ^]^ using bedtools^[^ ^71^ ^]^ (https://bedtools.readthedocs.io/en/latest/). Only PAS identified by DRS or ONT DRS were used for further analysis.

### DEPAS Analysis

DEPAS (Differentially Expressed Poly(A) Sites) were identified in *fpa* or *pcfs4* versus WT plants using the DESeq2 software^[^ ^72^ ^]^ (https://bioconductor.org/packages/release/bioc/html/DESeq2.html) with a threshold of fold change > 1.5 and *p*‐value < 0.05. To determine the gene transcription level, all PATs (Poly(A) Tags) of the gene transcripts were summed. DEPAS resulting from transcriptional level differences between *fpa* or *pcfs4* and WT plants were removed, filtered out DEPAS for which at least two biological replicates satisfied Diff = (PAS reads count in mutant / PAS reads count in WT) / (Gene reads count in mutant / Gene reads count in WT) > 1.2 or < 0.8. The filtered PAS (Poly(A) Sites) were used to calculate PAS usage, which was defined as the ratio of reads at one PAS relative to the total reads of the gene transcripts. Relative PAS usage was calculated as PAS usage (mutant) / PAS usage (WT). The relative PAS usage for each biological replicate was calculated individually, and the average usage from the three biological replicates was used in Figure 2.

### APA Event Analysis

Pre‐mRNAs with at least two poly(A) sites (PAS) were selected for alternative polyadenylation (APA) event analysis. The relative proximal/distal PAS usage ratio was calculated as: (proximal PAS usage [mutant]/ distal PAS usage [mutant])/ (proximal PAS usage [WT]/distal PAS usage [WT]).

UR‐APA was assessed by comparing all isoforms with PAS in upstream exon or intron regions (proximal PAS) versus all isoforms with PAS in the 3′ UTR (distal PAS) of the gene transcripts. Gene transcripts with a proximal/distal PAS ratio between 0.02 and 50 in WT were used for UR‐APA analysis. Lengthened or shortened transcripts in *fpa* or *pcfs4* were identified by a relative proximal/distal PAS usage ratio of < 0.8 or > 1.5, respectively.

3′ UTR‐APA was based on the two most abundant PAS isoforms in the 3′ UTR in the WT of the gene transcripts. Gene transcripts with the two PAS usage > 5% in at least two replicates of one sample, and with a relative PAS usage change of one PAS isoform in *fpa* or *pcfs4* versus WT > 50%, were used for 3′ UTR‐APA analysis. Lengthened or shortened 3′ UTRs in *fpa* or *pcfs4* compared to WT were identified by a relative proximal/distal PAS usage ratio of < 1 or > 1, respectively.

For 3′ extension events, up‐regulated differentially expressed PAS (DEPAS) that lie downstream of the 3′ UTR of the pre‐mRNA in *fpa* or *pcfs4* compared to WT were analyzed. Strand‐specific RNA‐Seq data were used to confirm that the reads mapping to the 3′ end extended from the 3′ UTR to the downstream PAS with increased usage in *fpa* or *pcfs4* compared to WT.

Scatter plots and box plots were generated using ggplot2 (https://ggplot2‐book.org).

### RT‐qPCR Analysis

Total RNA was isolated from 9‐day‐old plants (grown on 1/2 MS medium under long‐day conditions) using Trizol reagent (T9108, Takara). The RNA was then treated with RNase‐free DNase to remove any residual DNA. Three micrograms of DNA‐free total RNA were used for first‐strand cDNA synthesis with an oligo(dT) primer using the RevertAid First Strand cDNA Synthesis Kit (K1622, Thermo Fisher Scientific). Real‐time PCR was performed using Takara TB Green Premix Ex Taq on the QuantStudio 6 Flex Real‐Time PCR system (Applied Biosystems). *ACTIN* was used as an internal control, and the relative RNA abundance in *fpa*, *pcfs4*, or *fpa pcfs4* mutants was normalized to that in the wild type (WT). The primers used for detecting the expression levels of different mRNA isoforms are provided in Table S5 (Supporting Information).

### Tobacco Infiltrations and Fluorescence Resonance Energy Transfer (FRET)

The CDS (coding sequences) of *FPA*, *PCFS4*, or *CLPS3* driven by the 35S promoter and tagged with *GFP* or *RFP* (*mCherry*) were cloned into the pCAMBIA1300 vector. For truncated proteins, the following CDS regions were used: FPA_N_ (1‐440 aa), FPA_SPOC+C_ (441‐901 aa), PCFS4_N_ (1‐194 aa), PCFS4_IRD_ (195‐518 aa), and PCFS4_C_ (519‐808 aa).

*Agrobacterium* cultures (GV3101 strain) carrying the relevant constructs were grown overnight at 28 °C in YEB medium containing 25 mg L^−1^ rifampicin and 50 mg L^−1^ kanamycin to an OD600 of 1.0–1.2. The cultures were washed with infiltration buffer (10 mm MgCl_2_, 10 mm MES pH 5.7, and 200 µm acetosyringone), re‐suspended in the infiltration buffer, and gently shaken for 3–6 h, then diluted to an OD600 of 1.0. For FRET experiments, an equal amount of each culture was mixed. Fully expanded 3rd or 4th leaves from 4‐week‐old tobacco plants were infiltrated with these diluted and mixed *Agrobacterium* cultures using 1 mL syringes. Leaves were observed by confocal microscopy (Zeiss 780) 48 h after infiltration.

An acceptor photobleaching FRET assay was used to examine the interaction between the two indicated proteins. Bleaching of the acceptor fluorescence signal was performed using a 561 nm laser at maximum intensity for 15–30 s. Images from the GFP (donor, excitation 488 nm, emission 500–530 nm) and RFP (acceptor, excitation 561 nm, emission 600–630 nm) channels were captured before and after photobleaching. The fluorescence intensities of the donor and acceptor in pre‐ and post‐bleach images were determined. The FRET efficiency was calculated as: E_FRET_ = (I_Donor_^post^ – I_Donor_^pre^)/I_Donor_^post^, where I_Donor_ was fluorescence intensity of the donor. At least 8 cells were assayed to detect the interaction between the two tested proteins.

### Yeast Two Hybrid Assay

The coding sequences (CDS) of the tested proteins were cloned into the pGADT7 (AD) or pGBKT7 (BD) vectors. Transformation and yeast growth assays were performed as described in the Clontech Yeast Protocols Handbook. Briefly, the indicated two plasmids were co‐transformed into the yeast strain AH109 and plated on SD/‐Leu/‐Trp medium. The plates were incubated at 30 °C for 3 days upside‐down until colonies appeared. Fifteen colonies from the SD/‐Leu/‐Trp medium were re‐suspended in sterile water and re‐plated on SD/‐Leu/‐Trp/‐His/‐Ade (SD[‐LWHA]) medium to determine the interaction between the tested proteins.

For truncated proteins, the following CDS regions were used: FPA_N_ (1‐440 aa), FPA_SPOC+C_ (441‐901 aa), FPA_N+SPOC_ (1‐561 aa), FPA_C_ (562‐901 aa), FPA_SPOC_ (441‐561 aa), FPA_ΔSPOC_ (1‐440 + 562–901 aa), PCFS4_N_ (1‐194 aa), PCFS4_IRD_ (195‐518 aa) and PCFS4_C_ (519‐808 aa).

### Yeast Three Hybrid Assay

The coding sequences (CDS) of *FPA* or *CLPS3* were cloned into the MCS I site, and the CDS of *PCFS4* was cloned into the MCS II site of the pBridge (pB) vector (Clontech) to generate the *pB‐FPA‐PCFS4* and *pB‐CLPS3‐PCFS4* constructs, respectively. Transformation and yeast growth assays were performed as described in the Clontech Yeast Protocols Handbook. Briefly, the Y2H Gold yeast strain was activated four times on SD/‐Met medium. The constructs *AD‐CLPS3* / *pB‐FPA‐PCFS4* or *AD‐FPA* / *pB‐CLPS3‐PCFS4* were co‐transformed into Y2H Gold and plated on SD/‐Leu/‐Trp/‐Met medium. The plates were incubated at 30 °C for 3–4 days upside‐down until colonies appeared. Fifteen colonies from the SD/‐Leu/‐Trp/‐Met medium were re‐suspended in sterile water and re‐plated on SD/‐Leu/‐Trp/‐Met/‐His/AbA (SD[‐LWHM]+AbA) medium to determine the interaction between FPA and CLPS3 in the presence of PCFS4.

### Protoplast Transient Expression and Co‐Immunoprecipitation (Co‐IP) in Protoplast

Protoplasts were isolated from 4‐week‐old *Arabidopsis* leaves grown under a 10 h light/14 h dark photoperiod as described.^[^ ^73^ ^]^ Briefly, 0.5–1 mm leaf strips were cut from the middle part of well‐expanded leaves and transferred quickly and gently into the prepared enzyme solution (20 mm MES pH 5.7, 1.5% [wt/vol] cellulase R10, 0.4% [wt/vol] macerozyme R10, 0.4 m mannitol, 20 mm KCl, 10 mm CaCl_2_, and 0.1% BSA). The leaf strips were vacuum infiltrated for 30 min and digested in the dark for at least 3 h at room temperature. The enzyme/protoplast solution was then diluted with an equal volume of W5 solution (2 mm MES pH 5.7, 154 mm NaCl, 125 mm CaCl_2_, and 5 mm KCl), filtered through miracloth (475 855, Merck), and centrifuged at 100 *g*. The protoplasts were re‐suspended in W5 solution, incubated on ice for 30 min, and then re‐suspended at 2 × 10^5^ protoplasts/mL in MMG solution (4 mm MES pH 5.7, 0.4 m mannitol, and 15 mm MgCl_2_). The protoplasts were kept at room temperature and were ready for transformation.

The CDS of *FPA* with a *FLAG* tag and nos terminator, and the CDS of *PCFS4*, *CLPS3*, *CPSF100*, *CPSF30*, and *CstF64* with *MYC* tag and nos terminator, were cloned into the YNE vector, respectively. The plasmids were purified by density gradient centrifugation.

For co‐transformation, 400 µg of each plasmid in 400 µL ddH_2_O, 4 mL protoplasts in MMG solution, and 4.4 mL PEG/Ca^2+^ solution (40% PEG 4000, 0.2 m mannitol, and 0.1 m CaCl_2_) were mixed gently and completely. The mixture was incubated at room temperature for 8–15 min, diluted with 17.5 mL W5 solution, and centrifuged. The protoplasts were re‐suspended in 10 mL W5 solution and incubated for 12–18 h under weak light in the growth chamber at 22 °C.

After incubation, the protoplasts were collected by centrifugation at 80 *g* for 3 min, and total protein was extracted using 500 µL lysis buffer (50 mm Na_2_HPO_4_/NaH_2_PO_4_ pH 7.4, 150 mm NaCl, 0.2% Triton X‐100, 10% glycerol, 1% PMSF, and protease inhibitor cocktail). An 80 µL aliquot of the protein lysate was kept as input, and the remainder was incubated with 15 µL pre‐equilibrated anti‐FLAG M2 magnetic beads (M8823, Sigma) for 2 h at 4 °C. The FLAG‐tagged protein complex (bound to the magnetic beads) was collected using a magnetic stand and washed three times with lysis buffer. The complex was eluted with 1× SDS loading buffer at 100 °C for 10 min and probed with anti‐FLAG (F3165, Sigma) and anti‐MYC (M4439, Sigma) antibodies, respectively.

### Production of Anti‐FPA and Anti‐PCFS4 Antibodies

To produce polyclonal anti‐FPA and anti‐PCFS4 antibodies, the C‐terminus of FPA (441‐901 aa) and the 600–770 aa region of PCFS4, both tagged with a His tag, were expressed in *E. coli* (Rosetta strain). The recombinant proteins were purified using Ni‐NTA agarose beads (30 210, Qiagen) and used as antigens to immunize rabbits. The anti‐FPA and anti‐PCFS4 antibodies were then purified from the immune serum using protein A. The specificity and valence of the purified antibodies were assessed by Western blot using total protein extracts from wild type (WT) and mutant plants.

Anti‐FPA and anti‐PCFS4 antibodies were produced by ABclonal Technology. All animal experiments were approved by the Animal Ethics Committee of ABclonal Technology (Approval No. T2024(07)).

### Co‐IP in Plants

One gram of 12‐day‐old plants (grown on 1/2 MS medium under long‐day conditions) was ground into fine powder in liquid nitrogen. Total protein was extracted using 500 µL of IP buffer (50 mm Tris‐HCl pH 7.4, 150 mm NaCl, 0.1% Triton X‐100, 1 mm PMSF, and protease inhibitor cocktail). 80 µL of the protein lysate was kept as the input sample. The remaining lysate was incubated with 15 µL of pre‐equilibrated anti‐FLAG M2 (M8823, Sigma) or anti‐MYC (88 843, Pierce) magnetic beads for 2 h at 4 °C. The immunoprecipitated protein complex (bound to the magnetic beads) was collected using a magnetic stand and washed three times with IP buffer. The complex was then eluted with 1× SDS loading buffer at 100 °C for 10 min. The eluted proteins were probed with the following antibodies: anti‐FLAG (F3165, Sigma), anti‐MYC (M4439, Sigma), anti‐FPA (generated in this study). These antibodies were used to detect the presence of the respective proteins in the immunoprecipitated complexes, confirming their interactions.

### Immunoprecipitation followed by Mass Spectrometry (IP‐MS)

Six grams of 12‐day‐old WT, FPA‐MYCres, or PCFS4‐MYCres plants (grown on 1/2 MS medium under long‐day conditions) were cross‐linked in 1% formaldehyde solution (137 mm NaCl, 2.7 mm KCl, 10 mm Na_2_HPO_4_, 2 mm KH_2_PO_4_, and 1% formaldehyde). The plants were vacuum infiltrated for 4 min, repeated twice. Cross‐linking was stopped by adding glycine to a final concentration of 0.125 m and vacuum infiltrating for an additional 5 min. The materials were then washed with ddH_2_O. The plant materials were ground into fine powder in liquid nitrogen, suspended in 30 mL of Honda buffer (0.44 m Sucrose, 1.25% Ficoll, 2.5% Dextran T40, 20 mm Hepes KOH pH 7.4, 10 mm MgCl_2_, 0.5% Triton X‐100, 5 mm DTT, 1 mM PMSF, and protease inhibitor cocktail), and filtered through miracloth. Nuclei were collected by centrifugation at 2500 *g* for 5 min at 4 °C, washed three times with Honda buffer, and lysed in 800 µL of nuclei lysis buffer (50 mm Tris‐HCl pH 7.4, 150 mm NaCl, 1% Triton X‐100, 0.8% SDS, 1 mm PMSF, and protease inhibitor cocktail) by sonication (3 s on/6 s off at 200 W for 8 min, repeated twice) using a Scientz‐IID sonicator.

The lysate was centrifuged at 15000 *g* for 10 min at 4 °C. The supernatant was diluted 8‐fold with dilution buffer (50 mm Tris‐HCl pH 7.4, 150 mm NaCl, 1% Triton X‐100, 1 mm PMSF, and protease inhibitor cocktail) and incubated with 30 µL of pre‐equilibrated anti‐Myc magnetic beads (88 843, Pierce) for 3 h at 4 °C. The immunoprecipitated protein complex was collected using a magnetic stand and washed sequentially with: low‐salt wash buffer (50 mm Tris‐HCl pH 7.4, 150 mm NaCl, 1% Triton X‐100, 0.1% SDS), high‐salt wash buffer (50 mm Tris‐HCl pH 7.4, 500 mm NaCl, 1% Triton X‐100, 0.1% SDS), LiCl buffer (10 mm Tris‐HCl pH 7.4, 250 mm LiCl, 1% NP‐40, 0.5% sodium deoxycholate) and Tris buffer (10 mm Tris‐HCl pH 7.4).

The proteins co‐immunoprecipitated with FPA‐MYC or PCFS4‐MYC were eluted and reverse cross‐linked using 1× SDS loading buffer at 100 °C for 10 min. The samples were subjected to SDS‐PAGE, and the gel was stained with Coomassie brilliant blue. The light and heavy chains of the antibodies were cut out, the remaining gel was subjected to in‐gel digestion with trypsin, and the samples were submitted for mass spectrometry analysis using an LTQ Orbitrap Elite mass spectrometer (ThermoFisher Scientific). The data were analyzed using Thermo Scientific Proteome Discoverer software version 1.4, with the proteome sequences for *Arabidopsis thaliana* from TAIR11 used for database searching. The mass tolerance was set to 0.05 Da.

### RNA Immunoprecipitation (RIP)‐Seq and RIP‐PCR

Three grams of 12‐day‐old WT (Control) and GFP‐tag transgenic plants (grown on 1/2 MS medium under long‐day conditions) were cross‐linked in 1% formaldehyde solution as described in the IP‐MS section. Nuclei were extracted using Honda buffer (as mentioned in the IP‐MS section with 0.08 U mL^−1^ RNasin). The nuclei were lysed in 400 µL of nuclei lysis buffer (50 mm Tris‐HCl pH 7.4, 150 mm NaCl, 10 mM MgCl_2_,1% Triton X‐100, 0.8% SDS, 80 U mL^−1^ RNasin, 0.1 U µL^−1^ DNase I, 1 mm PMSF, and protease inhibitor cocktail) by sonication (3 s on/6 s off at 200 W for 10 min, repeated 3 times). After DNA digestion at 37 °C for 5 min, the nuclei lysate was centrifuged at 15000 *g* for 10 min at 4 °C. The supernatant was diluted 8‐fold with dilution buffer (50 mm Tris‐HCl pH 7.4, 150 mm NaCl, 10 mM MgCl_2_,1% Triton X‐100, 80 U mL^−1^ RNasin, 1 mm PMSF, and protease inhibitor cocktail) and pre‐cleared with 100 µL of pre‐equilibrated protein A/G magnetic beads (26 162, Pierce) at 4 °C for 1 h. The supernatant was transferred to a new tube, of which 200 µL was kept as input. The remaining supernatant was incubated with 12 µg of anti‐GFP antibodies (ab290, Abcam) at 4 °C overnight with gentle rotation. The protein‐RNA complex was captured by 25 µL of pre‐equilibrated protein A/G magnetic beads at 4 °C for 2 h. The beads were washed with low‐salt buffer, high‐salt buffer, LiCl buffer, and Tris buffer (as described in the IP‐MS section). The complex was eluted with 600 µL of elution buffer (200 mm NaCl, 1% SDS, 0.1 m NaHCO_3_, 0.08 U mL^−1^ RNasin) at 65 °C for 30 min, followed by reverse cross‐linking at 65 °C for an additional 60 min. After protein digestion with proteinase K (25 530 049, Invitrogen) at 45 °C for 1 h, the input and immunoprecipitated RNAs were extracted and precipitated with ethanol (including NaOAc and glycogen). The DNA‐free RNA (100‐300 bp) was subjected to sequencing on the Illumina Novaseq6000 platform (strand‐specific RNA sequencing, PE150, Novogene) or RT‐qPCR. Three independent biological replicates were conducted for the RIP‐Seq and RIP‐qPCR assays.

For RT‐qPCR, cDNA was synthesized using a random primer with the SuperScript III First‐Strand Synthesis System (18080‐051, Invitrogen). Real‐time PCR was performed using Takara TB Green Premix Ex Taq on the QuantStudio 6 Flex Real‐Time PCR system. The IPs were normalized to the input. The primers used are provided in Table S5 (Supporting Information).

### RIP‐Seq Data Analysis

Raw sequencing data were processed using fastp^[^ ^66^ ^]^ (https://github.com/OpenGene/fastp) to remove adaptors and low‐quality reads. Clean reads were then mapped to the *Arabidopsis* reference genome (Araport11) using HISAT2^[^ ^68^ ^]^ (http://daehwankimlab.github.io/hisat2/). Only uniquely aligned read pairs, extracted using samtools^[^ ^69^ ^]^ (http://www.htslib.org), were used for further analysis.

FPA binding sites were identified in FPA‐GFPres versus WT plants using the MACS2 peak calling software^[^ ^74^ ^]^ (https://github.com/taoliu/MACS/). MACS2‐normalized reads were used to identify FPA binding peaks with a threshold of *q* < 0.05. A total of 8233, 6741, and 5705 FPA binding sites were identified in the three independent biological replicates of FPA‐GFPres, respectively. Among these, 3840 common binding sites corresponding to 3017 unique genes were identified across all three replicates. No FPA binding sites were identified in FPA_ΔRRM_‐GFP/*fpa* using the same threshold.

The distribution of FPA binding sites around poly(A) sites was analyzed using bedtools^[^ ^71^ ^]^ (https://bedtools.readthedocs.io/en/latest/). MEME^[^ ^75^ ^]^ (https://meme‐suite.org/) was used to search for FPA binding elements in regions where FPA was bound compared to regions where it was not bound.

Heat maps of FPA binding sites were generated using deeptools^[^ ^76^ ^]^ (https://deeptools.readthedocs.io/).

### Purification of Recombinant Protein

The coding sequences (CDS) of FPA_RRM_ (1‐307 aa) and FCA_RRM_ (1‐343 aa) with a 6× His tag were cloned into the pMAL‐c2x vector. The constructs were transformed into *E. coli* Transetta (DE3, TransGen Biotech). Single colonies were cultured in 2YT medium containing ampicillin at 37 °C until the OD600 reached 0.6. Protein expression was induced with 1 mm IPTG (0487, Amresco) for 16 h at 16 °C. 100 mL of cells were harvested by centrifugation at 3000 *g* for 15 min. The cells were lysed in 7 mL of lysis buffer (50 mm NaH_2_PO_4_, 300 mm NaCl, 20 mm imidazole, 1 mm PMSF, pH adjusted to 8.0) using sonication. The lysate was centrifuged at 15000 *g* for 10 min, and the supernatant was incubated with 100 µL of Ni‐NTA agarose beads (30 210, Qiagen) at 4 °C for 1 h. The beads were washed with lysis buffer and stored in lysis buffer at 4 °C. Recombinant proteins were eluted with EMSA or ITC buffer containing 200 mm imidazole. The purity of the recombinant proteins was verified by 10% SDS‐PAGE and Coomassie blue staining.

### Electrophoretic Mobility Shift Assay (EMSA)

The MBP‐FPA_RRM_‐His, MBP‐FCA_RRM_‐His, or MBP‐His proteins were eluted from the Ni‐NTA agarose beads using EMSA buffer (10 mm Hepes‐KOH, 50 mm KCl, 0.05% Triton, 1 mm PMSF, and protease inhibitor cocktail) containing 200 mm imidazole. EMSA was performed using the LightShift Chemiluminescent RNA EMSA Kit (20 158, Invitrogen) as described. Briefly, biotin‐labeled RNAs were denatured by heating at 80 °C for 5 min and then cooled on ice. For each reaction, 2 nm biotin‐labeled probes and 0.5–4 µm purified protein were incubated in 1× binding buffer (10 mm HEPES‐KOH pH 7.3, 20 mm KCl, 1 mm MgCl_2_, 1 mm DTT, 5% glycerol, and 0.1 µg µL^−1^ tRNA) at room temperature for 30 min. The binding products were separated on a 6% native polyacrylamide gel at 4 °C and transferred to a positively charged nylon membrane at 4 °C. The protein bound to the biotin‐labeled RNA was visualized by chemiluminescence. The probe sequences used in the EMSA are listed in Table S5 (Supporting Information).

### Isothermal Titration Calorimetry (ITC) Measurements

The MBP‐FPA_RRM_‐His, MBP‐FCA_RRM_‐His, or MBP‐His proteins were eluted from the Ni‐NTA agarose beads using ITC buffer (10 mm HEPES‐KOH pH 8.0, 150 mm NaCl) containing 200 mm imidazole. The RNA was dissolved in the same buffer. All ITC measurements were performed at 25 °C using a MicroCal PEAQ‐ITC instrument (Malvern). The protein concentration in the cell (volume 300 µL) was 25 µm, and the RNA concentration in the injection syringe (volume 60 µL) was 400 µm. ITC titration experiments were conducted at 25 °C with 19 injections, each of 2 µL, with a 180‐s interval between each injection. The reference cell power was set to 10 µcal s^−1^. The titration data were analyzed using a one‐site binding model in the MicroCal PEAQ‐ITC analysis software provided by the manufacturer. The dissociation constant (K_d_) was automatically calculated and displayed in the software.

### Statistical Analyses

Data were presented as mean ± SD. The sample size (n) for each statistical analysis was indicated in the figure legends. Statistical analyses were performed using R.

Statistical significance between two groups for normally distributed continuous variables was assessed using unpaired, two‐tailed Student's *t*‐tests. For non‐normally distributed continuous or ordinal data, the Wilcoxon rank‐sum test was employed, whereas the Kolmogorov–Smirnov (K–S) test was used to evaluate differences in overall distributions. A *p*‐value < 0.05 was considered significant; experiment‐specific details were provided in the relevant figure legends.

## Conflict of Interest

The authors declare no competing interests.

## Author Contributions

Y.C., Y.G., Z.Y., and H.N. contributed equally to this work. L.M. and Y.C. designed the studies. Y.C., Y.L., X.S., Y.J., Q.Q.L., and L.M. analyzed and interpreted the data. L.M., Y.C., and Q.Q.L. wrote the manuscript. Y.C., Y.G., Z.Y., Q.L., H.X., C.Z., C.C., and Y.Z. performed the experiments. H.N., J.Y., D.Q., and X.X. performed PAT‐Seq, RNA‐Seq, and RIP‐Seq data analysis.

## Supporting information



Supporting Information

## Data Availability

All ssRNA‐Seq, PAT‐Seq, and RIP‐Seq data generated in this paper has been deposited at https://www.ncbi.nlm.nih.gov/bioproject. ssRNA‐Seq with the accession number PRJNA999439, PAT‐Seq with the accession number PRJNA999440, RIP‐Seq with the accession number PRJNA999469. The PAT‐Seq data for *cstf77*,^[^ ^77^ ^]^ *oxt6*,^[^ ^60^ ^]^ and *fip1* ^[^ ^78^ ^]^ are from the respective published papers, and the Helicos DRS and Nanopore DRS data for *fpa‐8* are from Parker et al., 2021.^[^ ^41^ ^]^
