# Supplementary material for: Polyadenylation Complex CFII Recognizes Downstream Cis‐element for Pre‐mRNA Polyadenylation Through Interaction with an RNA‐Binding Protein in Arabidopsis
Source: Adv Sci (Weinh). 2025 Aug 11;12(41):e04562. doi: 10.1002/advs.202504562 (PMC12591160; doi:10.1002/advs.202504562)
Supplement: Supplementary file 1 — Supporting Information [file ADVS-12-e04562-s001.docx]

**Supporting Information**

**Polyadenylation Complex CFII Recognizes Downstream Cis-element for Pre-mRNA Polyadenylation through Interaction with an RNA-binding Protein in Arabidopsis**

*Ying Cao^*^, Ying Guo, Zhibo Yu, Huajian Nie, Jing Yang, Dingfu Qiu, Qiyu Li, Xu Xin, Chang Cheng, Yan Li, Xudong Shang, Yuling Jiao,* *Qingshun Quinn Li ^*^, and Ligeng Ma^*^*


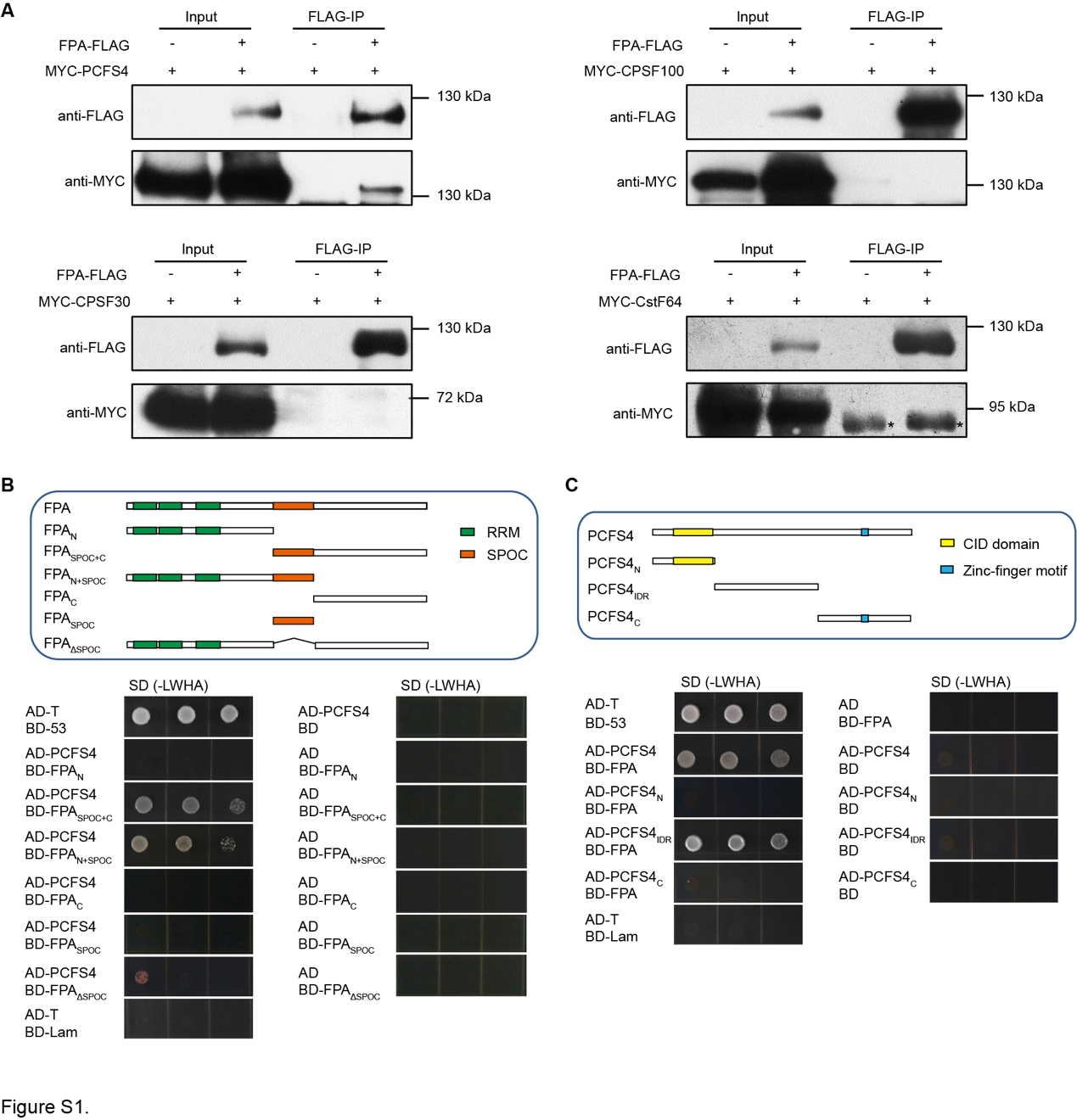


**Supplementary Figure 1. FPA and PCSF4 directly interacts each other via FPA_SPOC_ and PCFS4_IDR_ domain, respectively.**

A) Co-immunoprecipitation (Co-IP) demonstrates the association of FPA with PCFS4 in *Arabidopsis* protoplasts. The indicated proteins were expressed in Arabidopsis protoplasts, immunoprecipitated using M2 magnetic beads, and subsequently probed with the indicated antibodies. *Indicates non-specific binding.

B) Analysis of the interaction between full length PCFS4 and truncated FPA by yeast two-hybrid assay. Schematic diagrams of full-length and truncated FPA proteins are shown in the top panel.

C) Analysis of the interaction between full length FPA and truncated PCFS4 by yeast two-hybrid assay. Schematic diagrams of full-length and truncated PCFS4 proteins are shown in the top panel.

For yeast two-hybrid assay in B and C, positive control: AD-T/BD-53; negative control: AD-T/BD-Lam. The indicated two plasmids were co-transformed into the yeast reporter strain AH109, and the interaction of the tested two proteins was assessed by growth on SD/-LWHA.


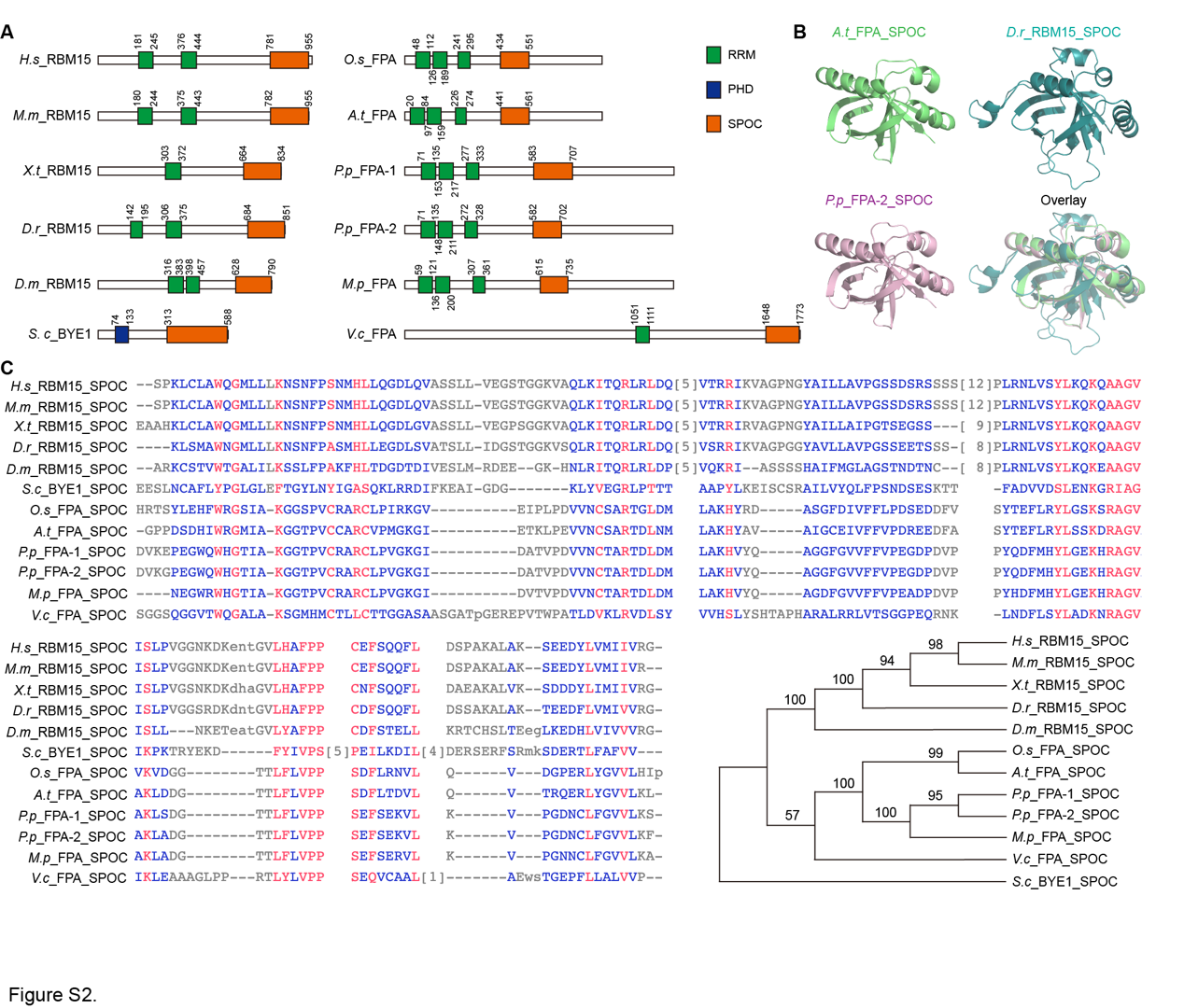


**Supplementary Figure 2. The SPOC domain of FPA is conserved among spen family proteins in eukaryotes.**

A) Domain architecture of spen family proteins in different species: *Homo sapiens* (*H.s*), *Mus musculus* (*M.m*), *Xenopus tropicalis* (*X.t*), *Danio rerio* (*D.r*), *Drosophila melanogaster* (*D.m*), *Saccaromyces cerevisiae* (*S.c*), *Oryza sativa* (*O.s*), *Arabidopsis thaliana* (*A.t*), *Physcomitrella patens* (*P.p*), *Marchantia polymorpha* (*M.p*) , *Volvox carteri* (*V.c*). (RRM) RNA recognition motif, (PHD) Plant homeodomain, (SPOC) Spen paralog and ortholog C-terminal domain.

B) The SPOC domain structures from *A.t*FPA, *P.p*FPA-1 and *D.r*RBM15 by AlphaFold2.

C) Multiple sequence alignment of the SPOC domain and the phylogenetic tree on the right bottom panel.

**
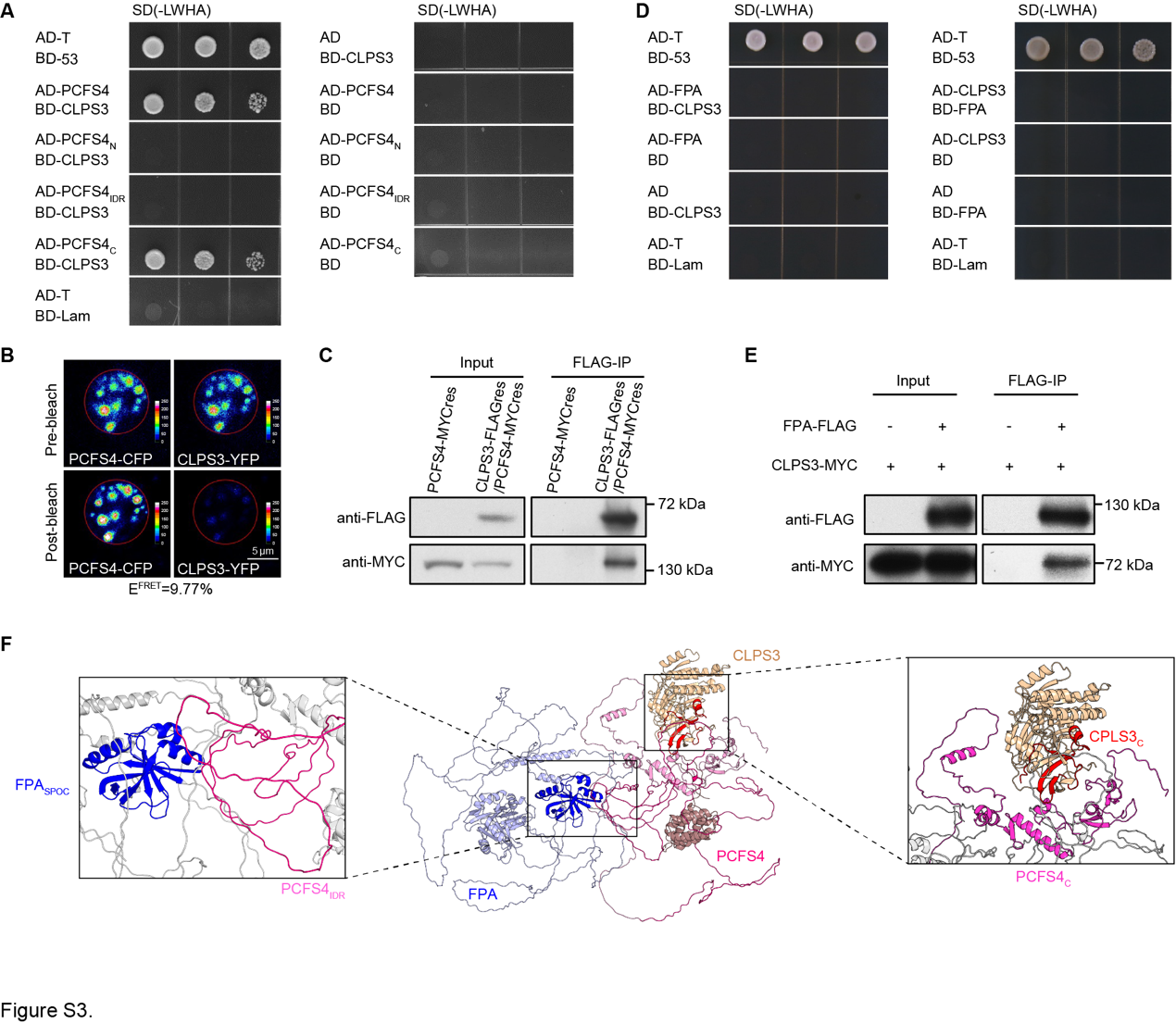
**

**Supplementary Figure 3. PCFS4 physically interacts with CLPS3 in *Arabidopsis*.**

A) Analysis of the interaction between PCFS4 and CLPS3 by yeast two-hybrid assay. Positive control: AD-T/BD-53; negative control: AD-T/BD-Lam. The two indicated plasmids were co-transformed into the yeast reporter strain AH109, and the interaction between the two tested proteins was evaluated by their growth on SD/-LWHA.

B) Analysis of the interaction between PCFS4 and CLPS3 in *N. benthamiana* cells by acceptor photobleaching FERT assay. Images of one nucleus from the GFP and RFP channels before and after photobleaching are shown. Different colors indicate the fluorescence intensity as indicated by the scale bars. The fluorescence intensities of the donor and acceptor in pre- and post-bleach images were determined. The FRET efficiency was calculated as: E_FRET_ = (I_Doner_^post^ - I_Doner_^pre^)/I_Doner_^post^, and ten cells were measured with FRET efficiencies all above 8%, averaging 9.2%.

C) Analysis of the association of PCFS4 with CLPS3 in *Arabidopsis* plants by Co-immunoprecipitation (Co-IP) assay. Total protein extracts from PCFS4-MYCres or PCFS4-MYCres/CLPS3-FLAGres plants were immunoprecipitated using M2 magnetic beads and subsequently probed with the indicated antibodies.

D) Detection of the direct interaction between FPA and CLPS3 by yeast two-hybrid assay. Positive control: AD-T/BD-53; negative control: AD-T/BD-Lam. The indicated two plasmids were co-transformed into the yeast reporter strain AH109, and the interaction of the tested two proteins was assessed by growth on SD/-LWHA.

E) Demonstration of the association of FPA with CLPS3 in *Arabidopsis* protoplast by Co-IP assay. The indicated proteins were expressed in Arabidopsis protoplasts, immunoprecipitated with M2 magnetic beads, and probed with the indicated antibodies.

F) The structures of FPA, PCFS4, and CLPS3, along with their interaction docking predicted by AlphaFord2, are shown here. The middle-central model illustrates PCFS4 (magenta) bridging FPA (blue) and CLPS3 (beige). Zoomed-in panels on the left and right highlight the specific interaction interfaces: the SPOC domain of FPA (FPA_SPOC_) interacts with the intrinsically disordered region of PCFS4 (PCFS4_IDR_), while the C-terminal region of PCFS4 (PCFS4_C_) contacts the C-terminal domain of CLPS3 (CLPS3_C_, red).


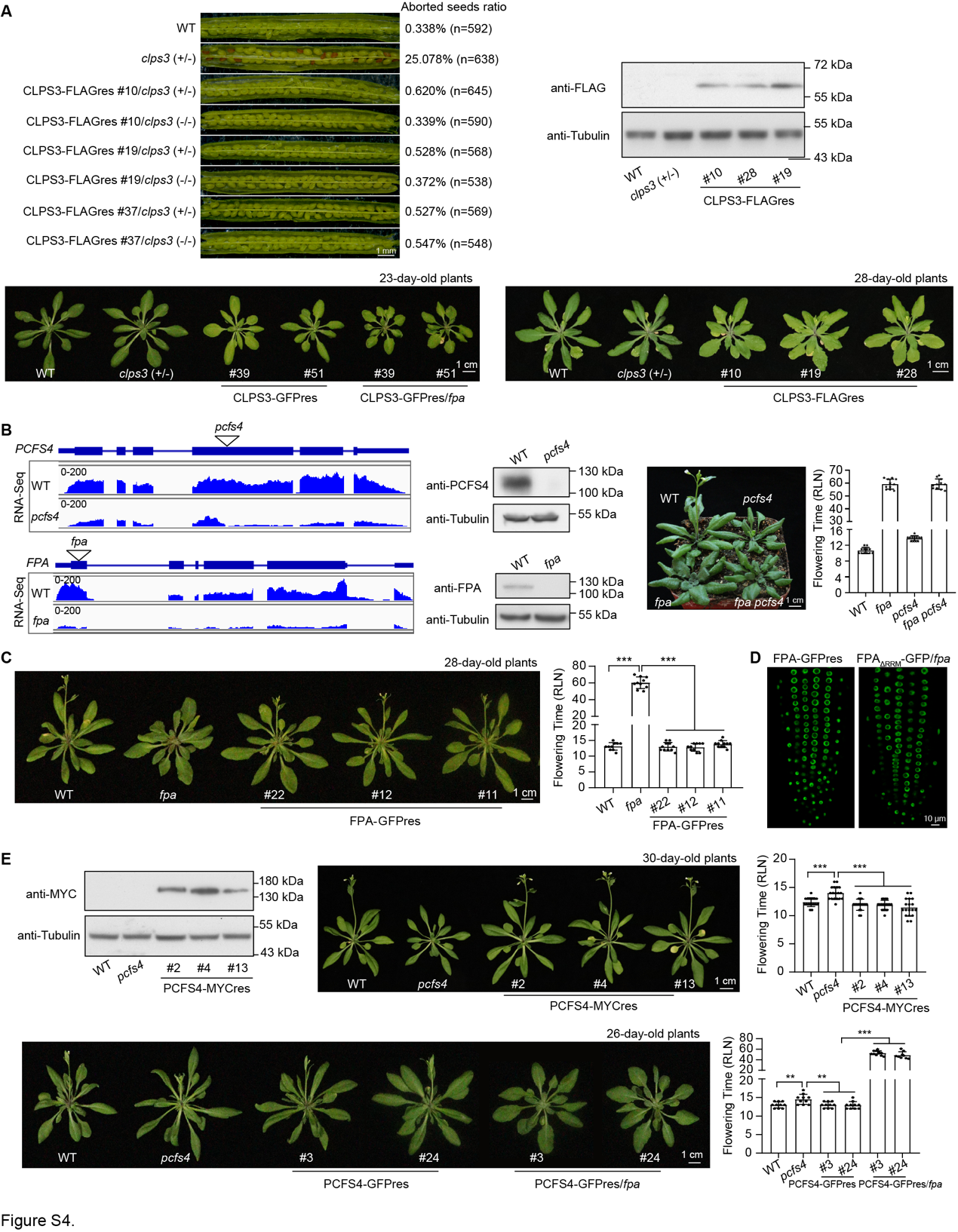


**Supplementary Figure 4. Characterization of *fpa*, *pcsf4* and *clps3* and corresponding transgenic lines.**

A) Complementation of *clps3* by CLPS3-FLAG and CLPS3-GFP. The ratio of aborted seeds in *clps3* heterozygote and the CLPS3-FLAG transgenic lines were calculated in the left-top panel. Western blot against anti-FLAG and anti-Tubulin antibodies in *clps3-1*(+/-) and CLPS3-FLAG rescued lines are shown in the right-top panel. The 23-day-old plants of CLPS3-GFP transgenic lines and 28-day-old plants of CLPS3-FLAG transgenic lines are shown in the left-bottom and right-bottom panel, respectively.

B) Characterization of *pcfs4* and *fpa* mutants. Gene structures and the RNA-Seq data exhibited by the Integrative Genomics Viewer of *PCFS4* and *FPA* are shown in the left panel. Western blot against anti-PCFS4 or anti-FPA antibodies in WT and the mutant are shown in the middle panel. The rosette leaf number (RLN) at bolting used as an indicator of flowering time is shown in the right panel. Data is presented as Mean ± SD, n≥10. Two-tailed Student’s t test, *p* <0.001 between the mutant and WT, while not significant between *fpa pcfs4* and *fpa*.

C) Complementation of *fpa* by FPA-GFP. 28-day-old plants are shown in the left panel.

D) Both full-length FPA and its truncated isoform FPA_ΔRRM_ are stably expressed and localized to the nucleus. Expression patterns were consistent across ≥10 independent transgenic lines for each construct, as shown in the figure.

E) Complementation of *pcfs4* by PCFS4-MYC and PCFS4-GFP, respectively. Western blot against anti-MYC and anti-Tubulin antibodies in *pcfs4* and PCFS4-MYC rescued lines are shown on the left panel. The 26-day-old PCFS4-GFP transgenic plants are shown in the left-bottom panel.

The rosette leaf number (RLN) at bolting was used as an indicator of flowering time in (C) and (E). Data is presented as Mean ± SD, n≥10. Two-tailed Student’s t test, *** *p* <0.001, ** *p* <0.01, not significant (ns) between the FPA or PCFS4 rescued lines and WT.


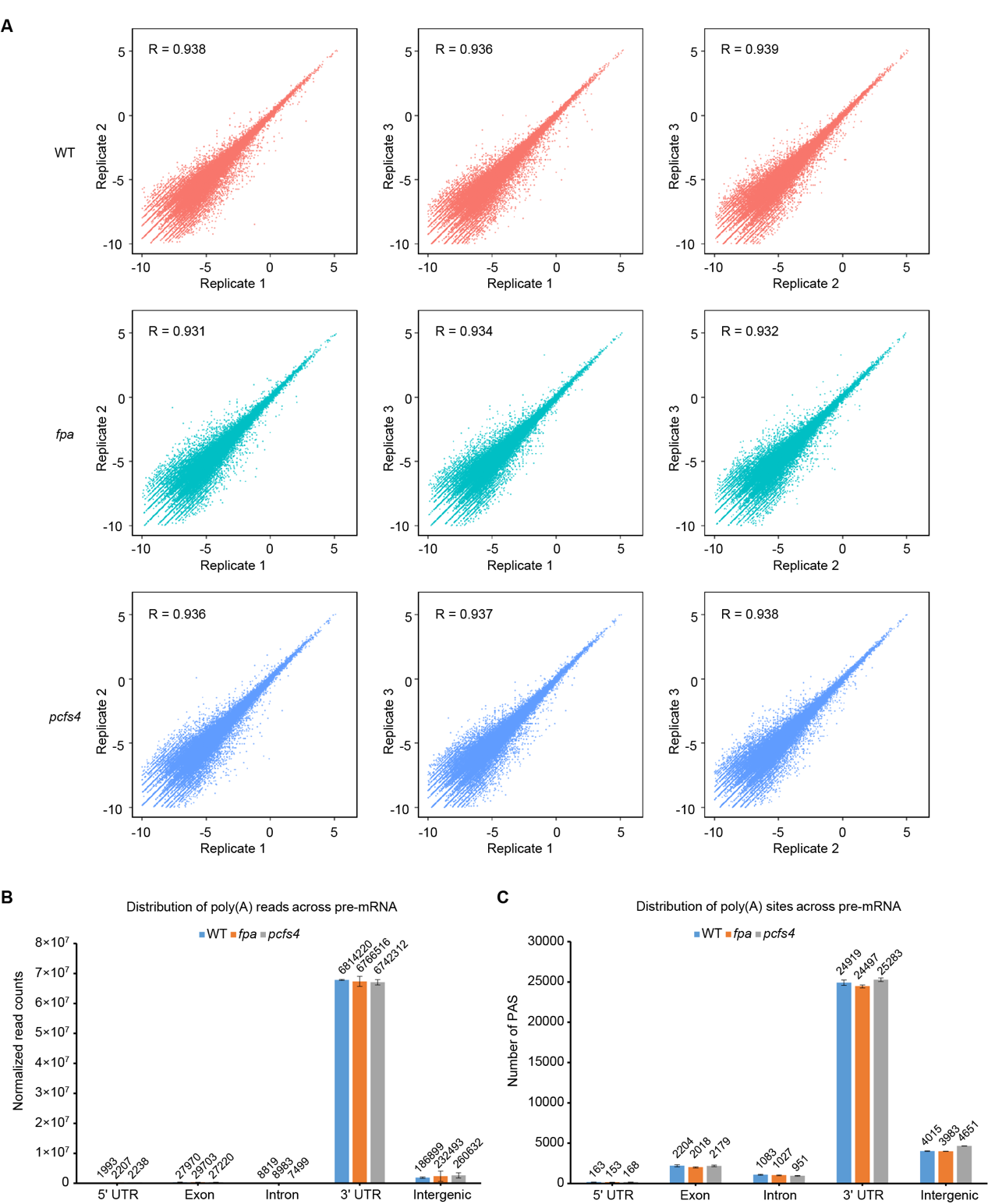


**Supplementary Figure 5.** PAT-Seq data demonstrated high reproducibility among the three independent biological replicates.

A) Pairwise correlation analysis among the three independent biological replicates from WT, *fpa*, and *pcfs4*, respectively. The reads per kilobase per million (RPKM) of each PAS in each biological replicate were used to calculate the Pearson’s correlation coefficient (R). The x-axis and y-axis represent RPKM values (log2) of the corresponding replicates.

B) Distribution of poly(A) reads among pre-mRNA structural features (PAT-Seq). Values are mean ± SD from three independent biological replicates; means are stated above each bar. Poly(A) read counts were normalized to the average sequencing depth across all samples.

C) Distribution of poly(A) sites (PAS) among pre-mRNA structural features (PAT-Seq). Values are mean ± SD from three independent biological replicates; means are stated above each bar.


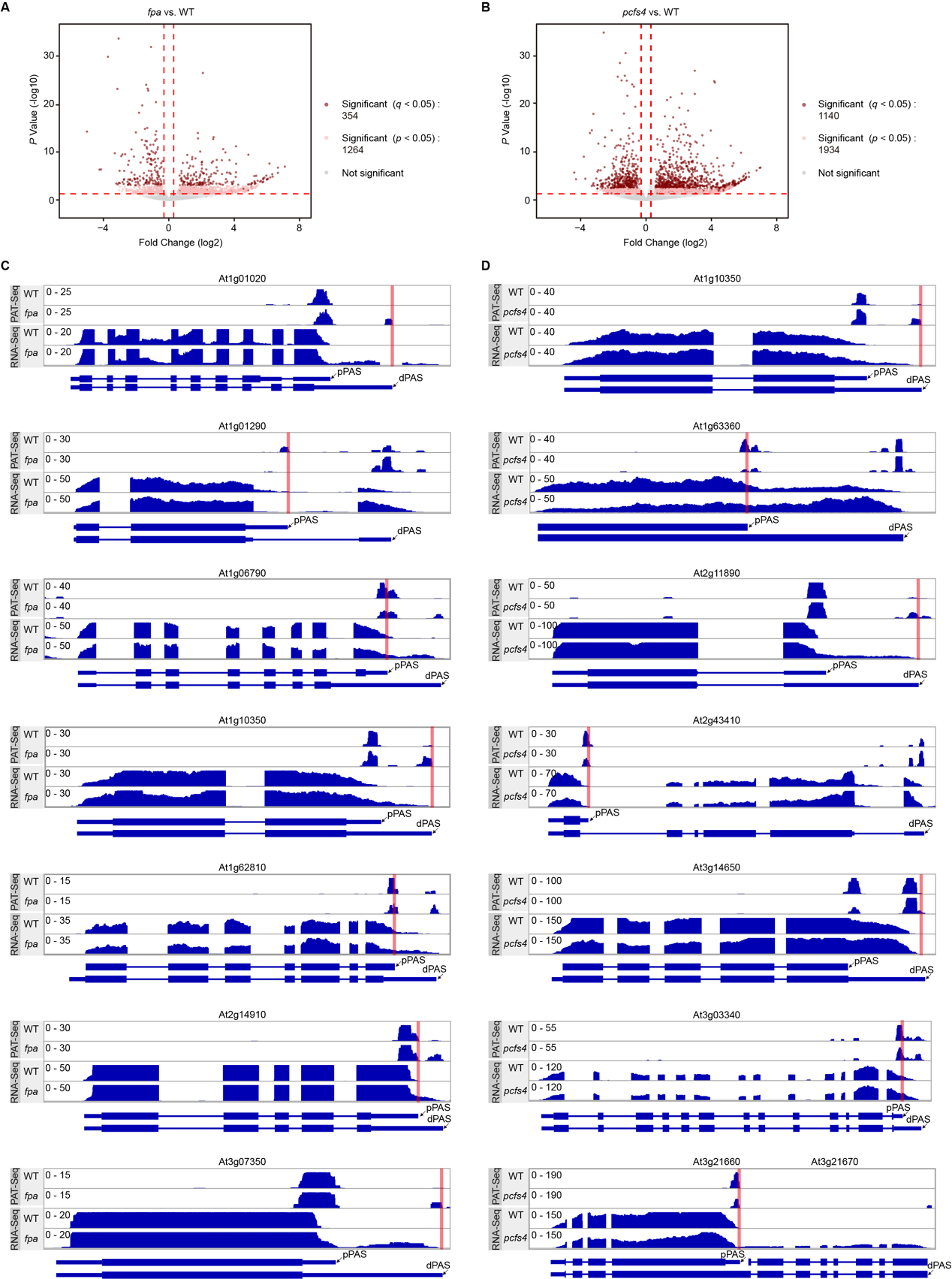


**Supplementary Figure 6. The identification of differentially expressed PAS in *fpa* vs. WT and *pcfs4* vs. WT.**

1. Volcano plot of differentially expressed poly(A) sites (DEPAS) in *fpa* vs. WT. Sites with |log₂FC| > 0.58 (fold change > 1.5) and *p* < 0.05, after filtering out transcription-driven changes, are highlighted: pink, *p* < 0.05 but *q* > 0.05; purple, *q* < 0.05. Using *p* < 0.05 as the cutoff, 1,618 DEPAS were identified; applying *q* < 0.05 reduced this to 354 DEPAS.
2. Volcano plot of differentially expressed poly(A) sites (DEPAS) in *pcfs4* vs. WT. After removing transcription-driven changes, sites with |log₂FC| > 0.58 (fold change > 1.5) and *p* < 0.05 are colored pink (*p* < 0.05 but *q* > 0.05) or purple (*q* < 0.05). In total, 3,704 DEPAS were identified with *p* < 0.05, and 1,140 with *q* < 0.05.

**C)** Seven DEPAS in *fpa* vs. WT that satisfy *p* < 0.05 but *q* > 0.05 were randomly selected and confirmed as reliable. Each DEPAS is indicated by a red line, with corresponding PAT-seq and RNA-seq tracks shown in the Integrative Genomics Viewer (IGV).

**D)** Seven DEPAS in *pcfs4* vs. WT that satisfy *p* < 0.05 but *q* > 0.05 were randomly selected and validated as reliable. Each DEPAS is marked with a red line, with corresponding PAT-seq and RNA-seq tracks displayed in the Integrative Genomics Viewer (IGV).

**
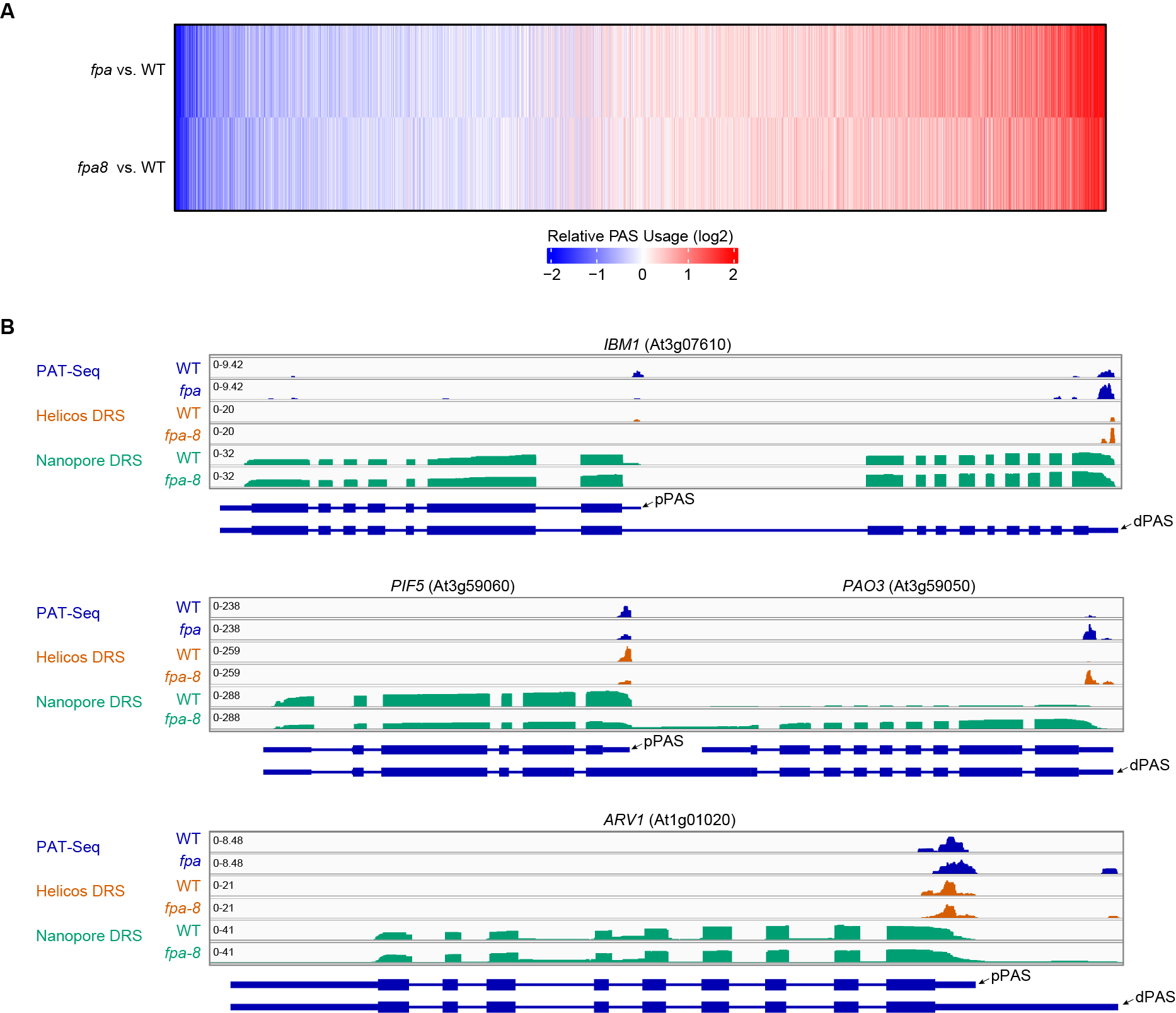
**

**Supplementary Figure 7. The DEPAS profile of *fpa* vs. WT in this study closely resemble those of the published *fpa-8* Helicos DRS dataset.**

A) Heat map of DEPAS in *fpa* vs. WT from this study and *fpa-8* vs. WT from a published study ^[41]^. The color scale indicates the fold change in relative PAS usage, with red for increased usage and blue for decreased usage in the mutant vs. WT.

B) Three representative mRNAs from three sequencing datasets were visualized in the Integrative Genomics Viewer (IGV). The datasets include PAT-Seq data from this study, Helicos DRS data and Nanopore DRS data from Parker et al., 2021 ^[41]^.

**
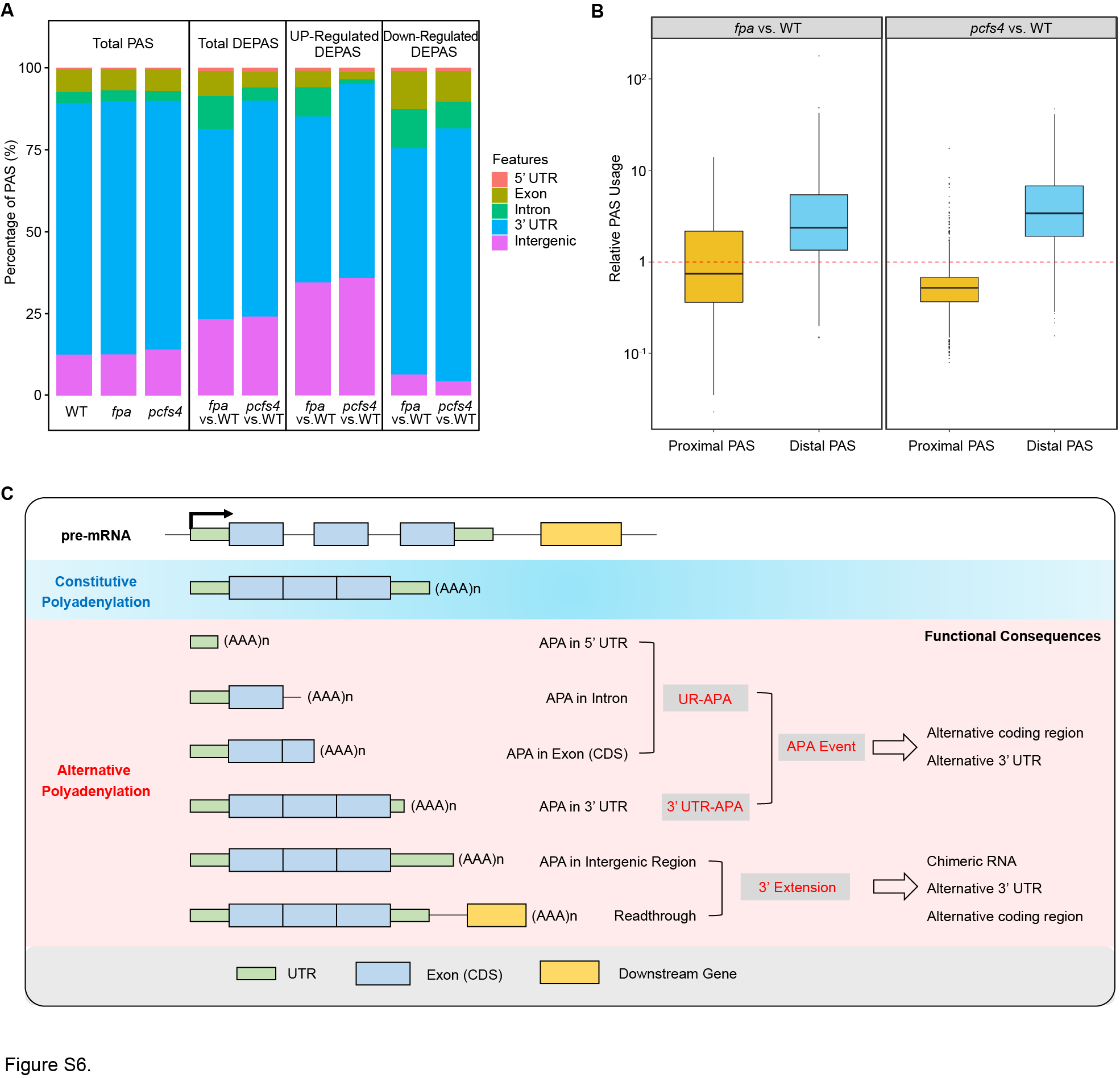
Supplementary Figure 8. Mutation in *FPA* or *PCFS4* leads to down-regulation of proximal PAS usage and up-regulation of distal PAS usage.**

A) Distribution of PAS and DEPAS in different structural regions of pre-mRNAs from wild type (WT), *fpa* and *pcfs4* plants.

B) The usages of proximal and distal PAS for DEPAS pre-mRNAs from *fpa* vs. WT and *pcsf4* vs. WT. Two most abundant PAS isoforms from each DEPAS pre-mRNAs from *fpa* vs. WT or *pcsf4* vs. WT were analyzed. The Kolmogorov-Smirnov (K-S) test was used to assess the significance of differences in the distribution of proximal and distal PAS usage for DEPAS pre-mRNAs shown in the figure, compared with all pre-mRNAs containing at least two poly(A) sites, *p*-value < 2.2e-16.

C) Schematic diagram of classification of alternative polyadenylation events. APA events include UR-APA and 3’ UTR-APA, UR-APA: alternative polyadenylation takes place upstream of the stop codon and leads to alternative coding region, 3’ UTR-APA: alternative polyadenylation takes place in the 3’ UTR and leads to alternative 3’ UTR; 3’ extension: alternative polyadenylation takes place in the 3’ intergenic region or the downstream gene and leads to 3’ extension.


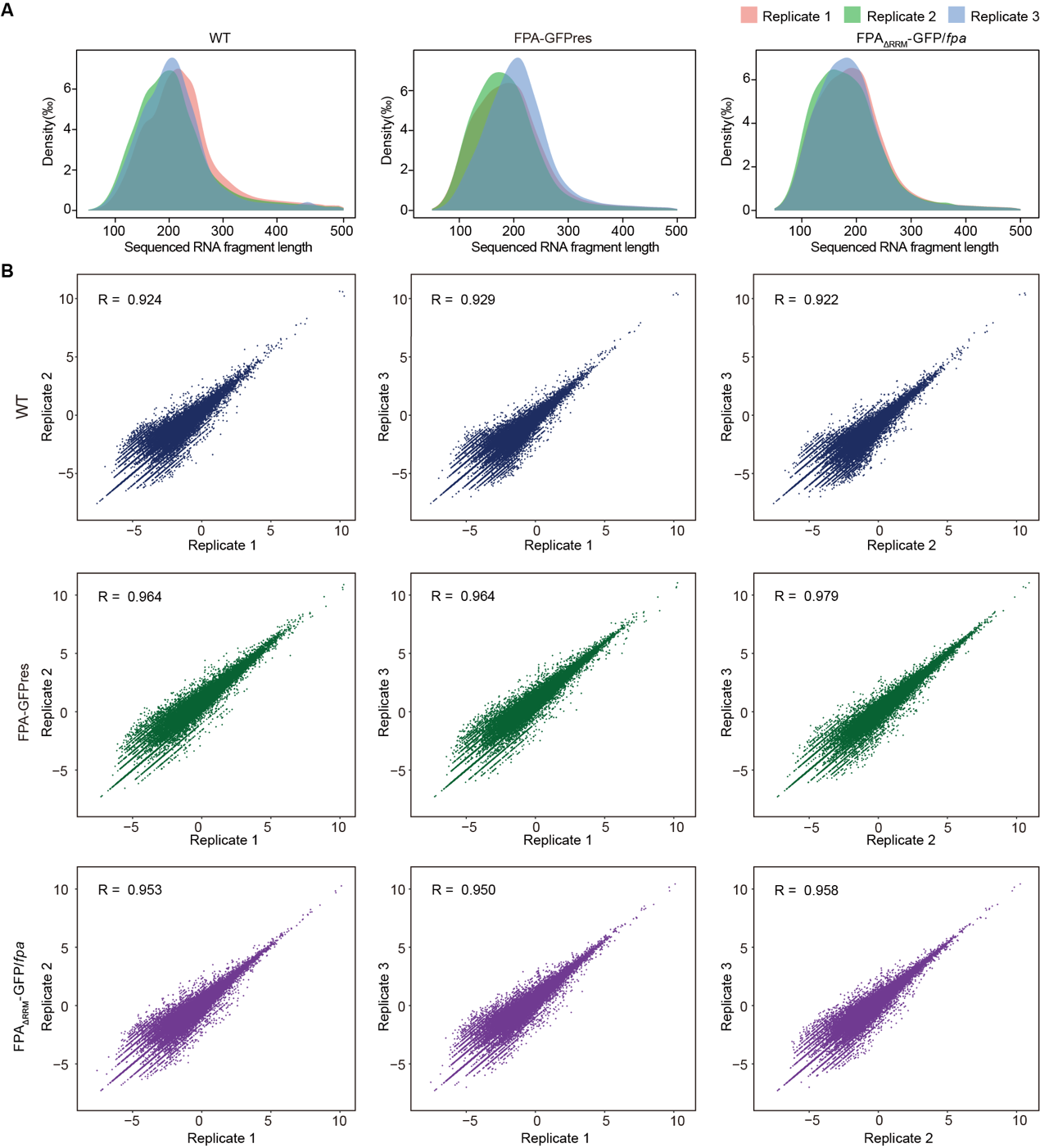


**Supplementary Figure 9. RIP-ssRNA-Seq** **data showed the high reproducibility among each of the three independent biological replicates.**

A) The RNA fragment size distribution for RIP-Seq samples from the three independent biological replicates of WT, FPA-GFPres and FPA_ΔRRM_-GFP/*fpa*, respectively.

B) Pairwise correlation analysis among each of the three independent biological replicates from WT，FPA-GFPres and FPA_ΔRRM_-GFP/*fpa*, respectively. The fragments per kilobase per million (FPKM) of each pre-mRNA in each biological replicate were used to calculate the Pearson’s correlation coefficient (R). The x-axis and y-axis were FPKM values (log2) of the corresponding replicates.


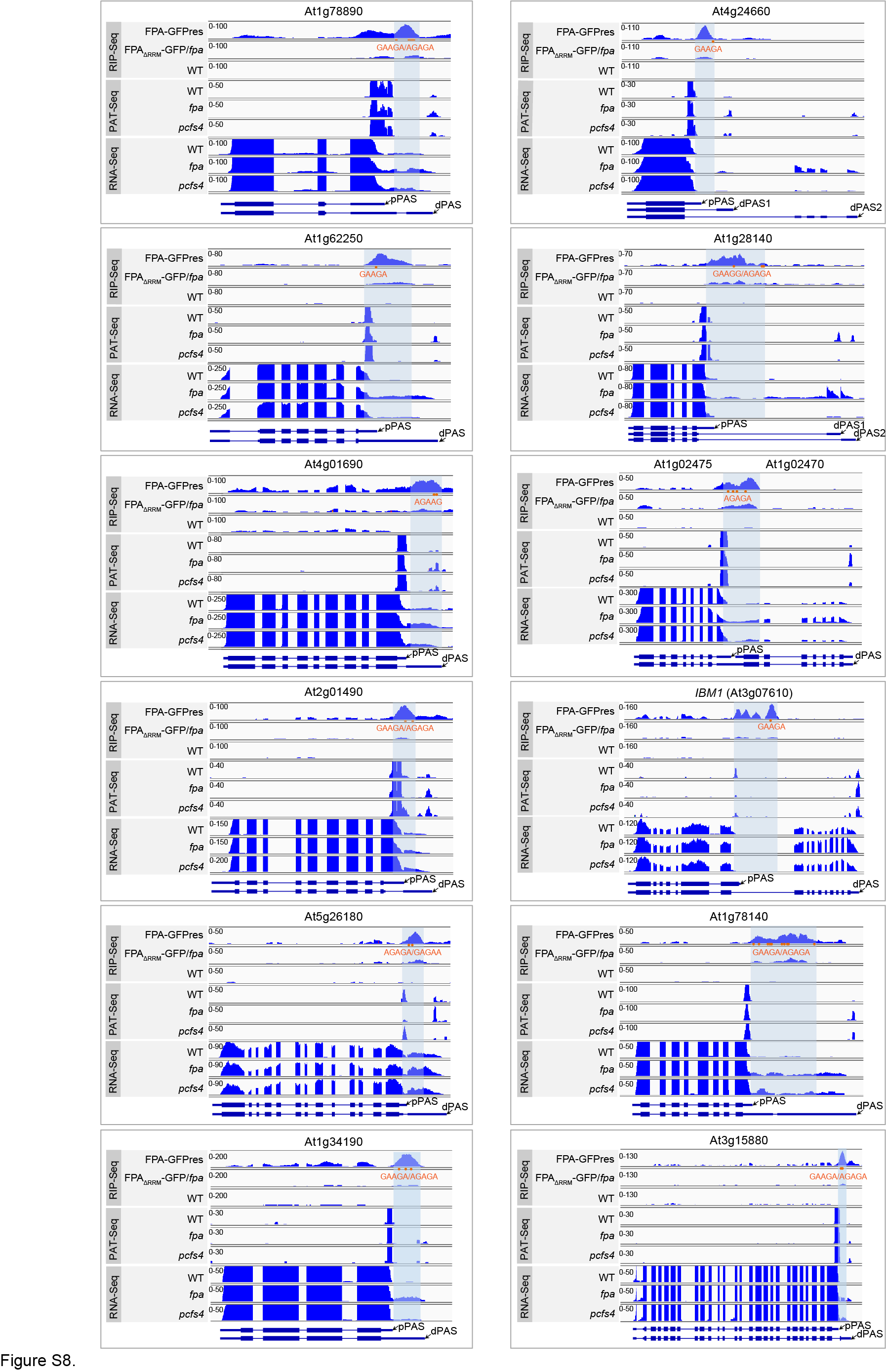


**Supplementary Figure 10. FPA binds to GA-rich element-containing region downstream of proximal PAS of target pre-mRNAs.**

RIP-Seq, PAT-Seq and RNA-Seq data exhibited by the Integrative Genomics Viewer show the binding of FPA to the downstream of the proximal PAS it promotes. pPAS: proximal poly(A) site, dPAS: distal poly(A) site, the orange dots indicate the position of GA-rich element.


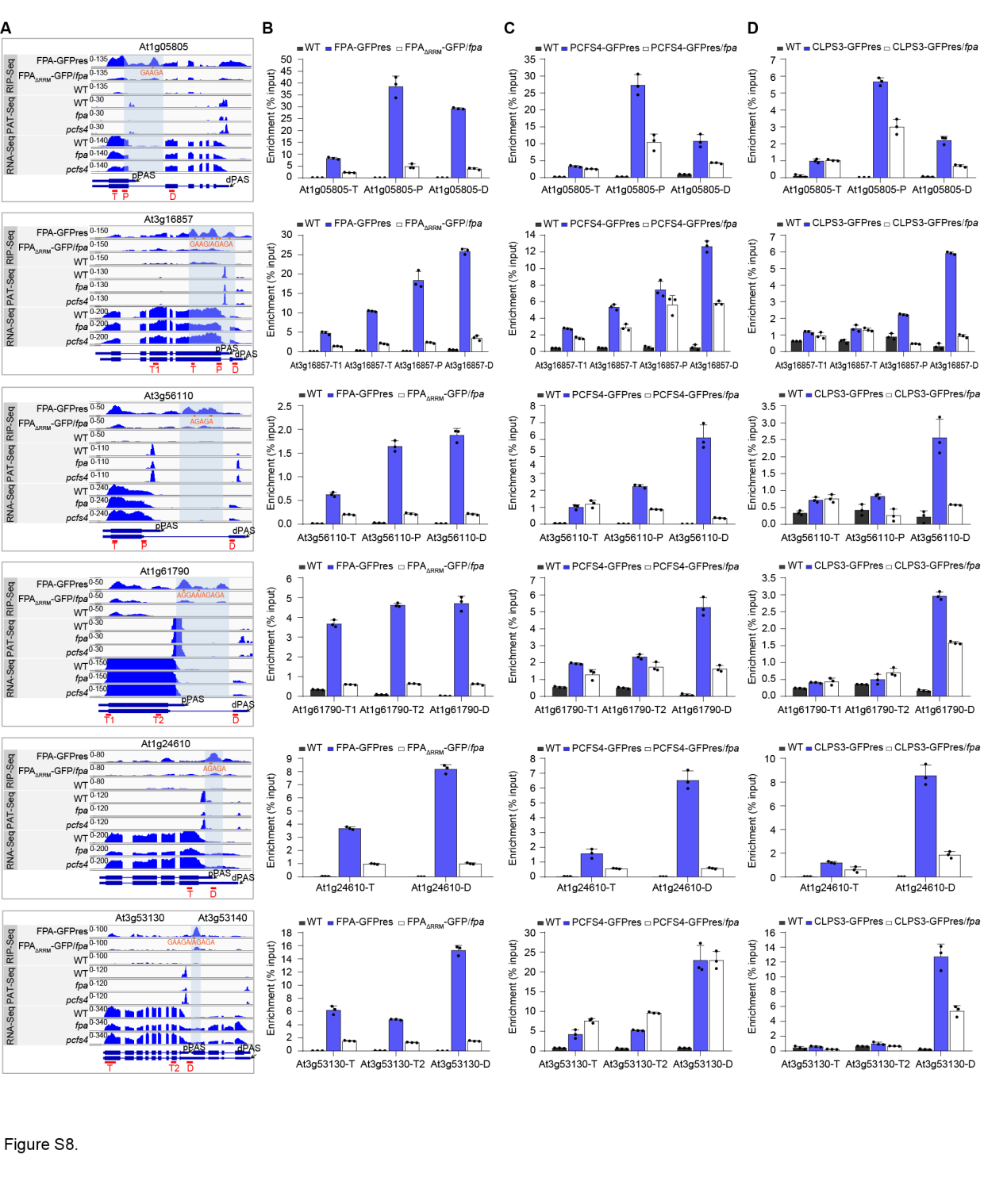


**Supplementary Figure 11. FPA bridges the binding of PCFS4 and CLPS3 to the GA-rich element-containing region downstream of proximal PAS of target pre-mRNA.**

A) FPA binds to the GA-rich element-containing region downstream of proximal PAS and is required for regulation of APA for representative pre-mRNAs in Figure 3 based on the RIP-Seq and PAT-Seq data exhibited by the Integrative Genomics Viewer. The red lines under the structure of pre-mRNA represents the position amplified mRNA by qPCR, the orange dots indicate the position of GA-rich element. Primer sequences are provided in Supplementary Table 5.

B-D) RIP-RT-qPCR analysis demonstrates the binding of FPA (B), PCFS4 (C) and CLPS3 (D) to the representative target pre-mRNAs, and the dependence of the RRM domain for FPA (B), or dependence of FPA for PCSF4 (C) and CLPS3 (D) for their binding to the targets. The IPs were normalized to the input. Three independent biological replicates were performed with similar results, and one representative result is shown. Data is presented as Mean + SD, n = 3 technical replicates.

**
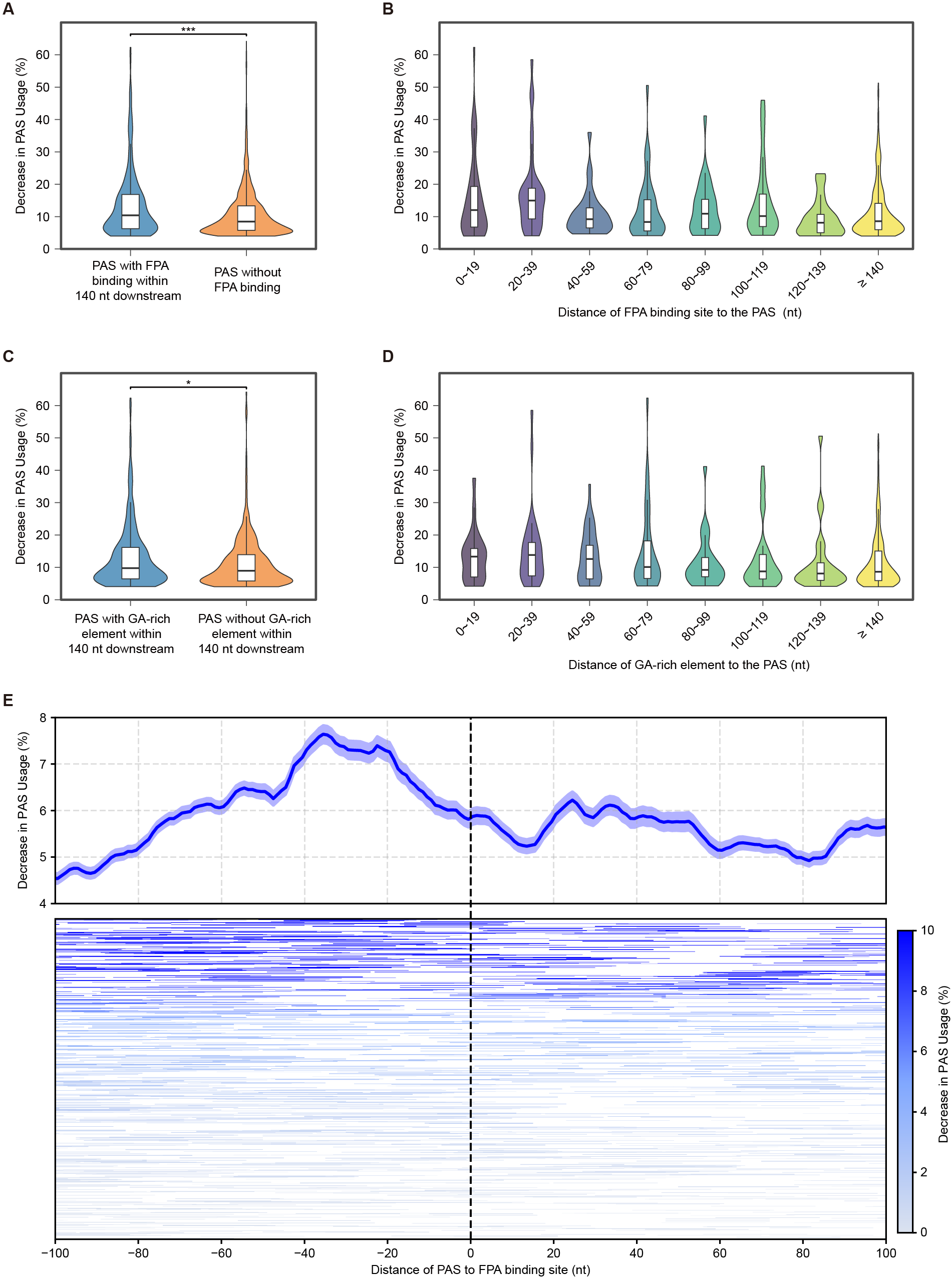
**

**Supplementary Figure 12.**  The impact of FPA binding and GA-rich elements on poly(A) site usage.

A) Violin plots illustrate the impact of FPA binding on poly(A) site usage. Poly(A) sites with reduced usage in *fpa* compared to WT were selected and divided into two groups: those with an FPA binding site within 140 nt downstream and those without. Wilcoxon rank sum test, *** *p* < 0.001.

B) The effect of FPA binding distance on poly(A) site usage. Poly(A) sites with reduced usage in *fpa* compared to WT and with FPA binding downstream were selected and grouped based on the distance between the poly(A) site and the nearest FPA binding peak. Usage changes were observed across these groups.

C) The impact of GA-rich elements on poly(A) site usage. Poly(A) sites with reduced usage in *fpa* compared to WT were selected and divided into two groups: those with a GA-rich element within 140 nt downstream and those without. Wilcoxon rank sum test, * *p* < 0.05.

D) The effect of GA-rich element distance on poly(A) site usage. Poly(A) sites with reduced usage in *fpa* compared to WT were selected and grouped based on the distance to the nearest GA-rich element located near the FPA binding peak. Usage changes were observed across these groups.

E) Effect of FPA binding on proximal poly(A) site usage. Top: Mean reduction (mean ± SEM) in PAS usage for all sites that show decreased utilization in *fpa* vs. WT within ±100 nt of the FPA binding site (dashed line, summit of FPA binding peak). Bottom: Heat map of every such PAS in the same window; each 24-nt row represents one site, with color intensity scaled to the magnitude of usage reduction.

**
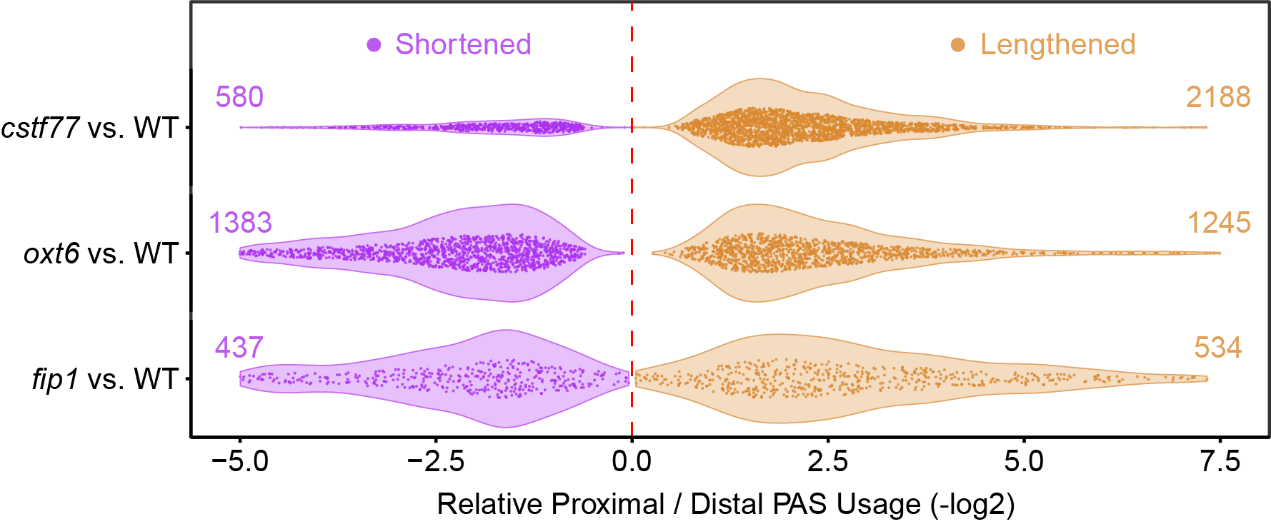
**

**Supplementary Figure 13.** Comparison of alternative polyadenylation events regulated by 3’ cleavage and polyadenylation factors.

Reanalysis of published PAT-Seq data for cstf77 ^[77]^, oxt6 (an AtCPSF30 mutant) ^[60]^, and fip1 ^[78]^ was performed. The two most abundant poly(A) sites (PAS) within each pre-mRNA were used for APA event analysis. Each dot represents an APA event, with the total number of lengthened and shortened transcripts indicated. Lengthened APA events (shift to distal PAS) are denoted in purple, and shortened APA events (shift to proximal PAS) are denoted in brown. PAS usage is calculated as the ratio of reads for one PAS isoform to the total reads of the mRNA. Relative PAS usage is defined as the ratio of PAS usage in the mutant to PAS usage in the wild type (WT).

**Supplementary Table 1. List of 3’ cleavage and polyadenylation factors identified by PCFS4-MYC affinity purification.**

| Identified Protein | Accession No. | Mol. Mass (kDa) | WT  IP1 | WT  IP2 | PCFS4-MYC  IP1 | | | PCFS4-MYC  IP2 | | |
| --- | --- | --- | --- | --- | --- | --- | --- | --- | --- | --- |
|  |  |  | MUP | MUP | MUP | PSM | XCorr | MUP | PSM | XCorr |
| PCFS4 | At4g04885 | 89.2 | 0 | 0 | 18 | 30 | 57.6 | 14 | 21 | 47.5 |
| CLPS3 | At3g04680 | 48.7 | 0 | 0 | 9 | 10 | 25.9 | 4 | 4 | 10.4 |
| FPA | At2g43410 | 99.5 | 0 | 0 | 1 | 1 | 1.6 | 1 | 1 | 2.3 |
| PCFS2 | At2g36480 | 90.8 | 0 | 0 | 2 | 2 | 7.1 | 2 | 2 | 5.8 |
| CPSF160 | At5g51660 | 158 | 0 | 0 | 1 | 1 | 3.4 | 0 | 0 | 0 |
| CPSF73a | At1g61010 | 77.3 | 0 | 0 | 1 | 1 | 2.0 | 0 | 0 | 0 |
| CPSF30 | At1g30460 | 28 | 0 | 0 | 1 | 1 | 4.1 | 0 | 0 | 0 |

MUP: Matched Unique Peptides; PSM: Peptide-Spectrum Match; XCorr: Cross-Correlation Score

**Supplementary Table 2. List of 3’ cleavage and polyadenylation factors identified by FPA-MYC affinity purification.**

| Identified Protein | Accession No. | Mol. Mass (kDa) | WT  IP1 | WT  IP2 | WT  IP3 | FPA-MYC  IP1 | | | FPA-MYC  IP2 | | | FPA-MYC  IP3 | | |
| --- | --- | --- | --- | --- | --- | --- | --- | --- | --- | --- | --- | --- | --- | --- |
|  |  |  | MUP | MUP | MUP | MUP | PSM | XCorr | MUP | PSM | XCorr | MUP | PSM | XCorr |
| FPA | At2g43410 | 99.5 | 0 | 0 | 0 | 21 | 45 | 85.4 | 28 | 61 | 105.0 | 26 | 56 | 103.1 |
| PCFS4 | At4g04885 | 89.2 | 0 | 0 | 0 | 3 | 3 | 9.5 | 3 | 5 | 10.4 | 3 | 4 | 9.9 |
| PCFS2 | At2g36480 | 90.8 | 0 | 0 | 0 | 6 | 6 | 18.5 | 11 | 11 | 33.9 | 11 | 13 | 35.7 |
| CLPS3 | At3g04680 | 48.7 | 0 | 0 | 0 | 1 | 1 | 3.2 | 4 | 4 | 11.0 | 5 | 5 | 13.7 |
| CPSF100 | At5g23880 | 82.1 | 0 | 0 | 0 | 3 | 4 | 10.7 | 8 | 10 | 28.9 | 6 | 8 | 22.4 |
| FY | At5g13480 | 72.2 | 0 | 0 | 0 | 4 | 4 | 12.3 | 4 | 4 | 12.2 | 4 | 4 | 14.2 |
| CPSF160 | At5g51660 | 158 | 0 | 0 | 1 | 4 | 4 | 9.8 | 8 | 8 | 25.8 | 12 | 12 | 36.8 |
| CPSF73a | At1g61010 | 77.3 | 0 | 0 | 0 | 1 | 1 | 3.3 | 9 | 10 | 30.9 | 10 | 11 | 30.1 |
| CPSF30 | At1g30460 | 28 | 0 | 0 | 0 | 1 | 1 | 3.4 | 3 | 4 | 9.8 | 3 | 4 | 10.5 |
| AtFIP1  /AtFIPS5 | At5g58040 | 133.4 | 0 | 0 | 0 | 3 | 3 | 8.2 | 2 | 2 | 7.2 | 6 | 6 | 16.5 |
| CSTF64 | At1g71800 | 50.1 | 0 | 0 | 0 | 3 | 3 | 8.7 | 5 | 5 | 13.6 | 6 | 6 | 19.7 |
| CSTF77 | At1g17760 | 82.6 | 0 | 0 | 0 | 1 | 1 | 3.6 | 2 | 2 | 5.5 | 2 | 2 | 5.9 |
| ESP1/  CstF64L | At1g73840 | 41.8 | 0 | 0 | 0 | 1 | 1 | 3.5 | 4 | 5 | 14.4 | 3 | 3 | 12.4 |
| SYM/ESP4 | At5g01400 | 159.4 | 0 | 0 | 0 | 2 | 3 | 7.5 | 8 | 10 | 23.8 | 8 | 8 | 23.2 |
| CFIS2/  CFIm25 | At4g25550 | 22.8 | 0 | 0 | 0 | 1 | 1 | 2.2 | 1 | 2 | 3.3 | 1 | 2 | 3.1 |
| PAPS1 | At1g17980 | 66 | 0 | 0 | 0 | 1 | 1 | 3.1 | 2 | 2 | 6.9 | 0 | 0 | 0 |
| Pab1 | At5g51120 | 25.7 | 0 | 0 | 0 | 2 | 2 | 7.3 | 2 | 2 | 6.6 | 2 | 2 | 6.8 |

MUP: Matched Unique Peptides; PSM: Peptide-Spectrum Match; XCorr: Cross-Correlation Score

**Supplementary Table 3. Summary of DEPASs in *fpa* vs. WT and *pcfs4* vs. WT in different** **structural regions of pre-mRNA.**

| Groups | Regions | Up-regulated DEPAS | | Down-regulated DEPAS | |
| --- | --- | --- | --- | --- | --- |
|  |  | Events | Frequency (%) | Events | Frequency (%) |
| *fpa* vs.  WT | Total | 954 | 100.00 | 664 | 100.00 |
|  | 5’ UTR | 8 | 0.84 | 6 | 0.90 |
|  | Exon | 44 | 4.61 | 67 | 10.11 |
|  | Intron | 77 | 8.07 | 70 | 10.56 |
|  | 3’ UTR | 443 | 46.44 | 402 | 60.48 |
|  | Intergenic | 302 | 31.66 | 37 | 5.58 |
| *pcfs4* vs.  WT | Total | 1,851 | 100.00 | 1,223 | 100.00 |
|  | 5’ UTR | 22 | 1.19 | 10 | 0.82 |
|  | Exon | 39 | 2.11 | 97 | 7.93 |
|  | Intron | 26 | 1.40 | 84 | 6.87 |
|  | 3’ UTR | 1,043 | 56.35 | 806 | 65.90 |
|  | Intergenic | 629 | 33.98 | 45 | 3.68 |

**Supplementary Table 4. Summary of the APA events in *fpa* vs. WT and *pcfs4* vs. WT.**

| Event Type | | *fpa* vs. WT | | *pcfs4* vs. WT | |
| --- | --- | --- | --- | --- | --- |
|  |  | Events | Frequency (%) | Events | Frequency (%) |
| Total APA Events | | 744 | 100.00 | 1551 | 100.00 |
| UR-APA | Total | 174 | 23.39 | 184 | 11.86 |
|  | Lengthened transcripts | 101 | 13.58 | 134 | 8.64 |
|  | Shortened transcripts | 73 | 9.81 | 50 | 3.22 |
| 3’ UTR-APA | Total | 244 | 32.80 | 913 | 58.87 |
|  | Lengthened transcripts | 153 | 20.56 | 897 | 57.83 |
|  | Shortened transcripts | 91 | 12.23 | 16 | 1.03 |
| 3’ extension |  | 326 | 43.82 | 454 | 29.27 |
|  | Lengthened transcripts |  |  |  |  |
|  |  |  |  |  |  |

**Supplementary Table 5. Oligos used in this work.**

| RNA | SOURCE/REF |
| --- | --- |
| **GU-rich probe** (used in REMSA and ITC): 5’ -GUUGUUUUGUUUGUUGUUUUGUUU - 3’ | Tian *et al*., 2019 |
| **GU-rich probe** (used in REMSA): 5’ - Biotin GUUGUUUUGUUUGUUGUUUUGUUU Biotin - 3’ | Tian *et al*., 2019 |
| **GA-rich probe** (used in REMSA and ITC): 5’ -GAAGAGAAGAAGAGAAGAAGAGAA - 3’ | Rui Biotech oligo |
| **GA-rich probe** (used in REMSA): 5’ - Biotin GAAGAGAAGAAGAGAAGAAGAGAA Biotin - 3’ | Rui Biotech oligo |
| **UC-rich probe** (used in REMSA): 5’ -UUCUCUUCUUCUCUUCUUCUCUUC - 3’ | Rui Biotech oligo |
| **UC-rich probe** (used in REMSA): 5’ - Biotin UUCUCUUCUUCUCUUCUUCUCUUC Biotin - 3’ | Rui Biotech oligo |
| DNA Oligonucleotides | SOURCE/REF |
| **AT1G08230-TF** (PCR primer for RIP) 5’ - GGCAGCCCATCCTAAGAACTTG - 3’ | Rui Biotech oligo |
| **AT1G08230-TR** (PCR primer for RIP) 5’ - CATGGTGGCACTGCGGATTC - 3’ | Rui Biotech oligo |
| **AT1G08230-PF** (PCR primer for RIP and qRT) 5’ - GTCGCAGCTTTGATTATAGGAAAGAG - 3’ | Rui Biotech oligo |
| **AT1G08230-PR** (PCR primer for RIP and qRT) 5’ - CTCGAACACCACGCTTCTCTC - 3’ | Rui Biotech oligo |
| **AT1G08230-DF** (PCR primer for RIP and qRT) 5’ - GAGTGAAAGGATGGGAATTGTGCC - 3’ | Rui Biotech oligo |
| **AT1G08230-DR**(PCR primer for RIP and qRT) 5’ - GTACTTGGTAGTGCAGCCAAATGG - 3’ | Rui Biotech oligo |
| **AT1G05805-TF** (PCR primer for RIP) 5’ - GCTATCAACCGTCGTCGGATAAC - 3’ | Rui Biotech oligo |
| **AT1G05805-TR** (PCR primer for RIP) 5’ - CTGTTTCTGTCCGTCGGATGAG - 3’ | Rui Biotech oligo |
| **AT1G05805-PF** (PCR primer for RIP and qRT) 5’ - GATCTTACTCTCTCGCTAGACAAC - 3’ | Rui Biotech oligo |
| **AT1G05805-PR** (PCR primer for RIP and qRT) 5’ - ATATCTCATCACAGTTACTATTCTTGAG - 3’ | Rui Biotech oligo |
| **AT1G05805-DF** (PCR primer for RIP and qRT) 5’ - ACTCTCTGGCTCGGATCAACG - 3’ | Rui Biotech oligo |
| **AT1G05805-DR** (PCR primer for RIP and qRT) 5’ - CTATCGAACCGGAACCGTCATC - 3’ | Rui Biotech oligo |
| **AT3G16857-T1F** (PCR primer for RIP) 5’ - GGAGAAGTGAATGGAGTGTACCG- 3’ | Rui Biotech oligo |
| **AT3G16857-T1R** (PCR primer for RIP) 5’ - ACCACCACCACCTCCTCTATG- 3’ | Rui Biotech oligo |
| **AT3G16857-TF** (PCR primer for RIP) 5’ - CCCAACAGCAACCACAGATGAAC - 3’ | Rui Biotech oligo |
| **AT3G16857-TR** (PCR primer for RIP) 5’ – CACTATTTCCAGCTCGAACAGCAG - 3’ | Rui Biotech oligo |
| **AT3G16857-PF** (PCR primer for RIP and qRT) 5’ – ACGAGCAGTATAGTAATCAAGAAG - 3’ | Rui Biotech oligo |
| **AT3G16857-PR** (PCR primer for RIP and qRT) 5’ – TCAGATTATGCTTCTATATAAGTTTGAC - 3’ | Rui Biotech oligo |
| **AT3G16857-DF** (PCR primer for RIP and qRT) 5’ –GTAGTCTGCATGAAATTTTAAATGGC - 3’ | Rui Biotech oligo |
| **AT3G16857-DR** (PCR primer for RIP and qRT) 5’ –AGCCTCACTACATAAATGAGGAAC - 3’ | Rui Biotech oligo |
| **AT5G13890-TF** (PCR primer for RIP) 5’ - CGAGAGGATCGGAGTAGAAGGAG - 3’ | Rui Biotech oligo |
| **AT5G13890-TR** (PCR primer for RIP) 5’ – GTCACCGGAAACAACCACCAC - 3’ | Rui Biotech oligo |
| **AT5G13890-PF** (PCR primer for RIP and qRT) 5’ - AGTTCCAAAAGAGTGACTGAGAGC - 3’ | Rui Biotech oligo |
| **AT5G13890-PR** (PCR primer for RIP and qRT) 5’ – AGATGAAGTCGTTGACAGGCAC - 3’ | Rui Biotech oligo |
| **AT5G13890-DF** (PCR primer for RIP and qRT) 5’ - CCACTAAGGTTTCACCATTCCCTC- 3’ | Rui Biotech oligo |
| **AT5G13890-DR** (PCR primer for RIP and qRT) 5’ – GCACTGGCCCAGATATGTCTTG - 3’ | Rui Biotech oligo |
| **AT3G56110-TF** (PCR primer for qRT) 5’ - GATCGATGAGCTCCGTCCATG - 3’ | Rui Biotech oligo |
| **AT3G56110-TR** (PCR primer for qRT) 5’ - GCAAGCTGTTCAATCTCAGCC - 3’ | Rui Biotech oligo |
| **AT3G56110-PF** (PCR primer for RIP and qRT) 5’ - AGAGCTTCGTTACGAATCTACATTCC - 3’ | Rui Biotech oligo |
| **AT3G56110-PR** (PCR primer for RIP and qRT) 5’ - CTCAGGGAGAGTCTAAGAGCTGG - 3’ | Rui Biotech oligo |
| **AT3G56110-DF** (PCR primer for qRT) 5 ’- CTTCGTTTAATGCGTCGGAGC - 3’ | Rui Biotech oligo |
| **AT3G56110-DR** (PCR primer for qRT) 5’ - GTCCCACAAATATAGCCGTTAGG - 3’ | Rui Biotech oligo |
| **AT1G61790-T1F** (PCR primer for RIP) 5’ - ACTATCATCCACATTCGCAGCTTC - 3’ | Rui Biotech oligo |
| **AT1G61790-T1R** (PCR primer for RIP) 5’ - CCTGATTCGCTTGTTGATCTGAGAG - 3’ | Rui Biotech oligo |
| **AT1G61790-T2F** (PCR primer for RIP) 5’ - GTGTTGCTGGTACAATGCACAAC - 3’ | Rui Biotech oligo |
| **AT1G61790-T2R** (PCR primer for RIP) 5’ - CAGATCCTTGGTAGAAAAACACAAGC - 3’ | Rui Biotech oligo |
| **AT1G61790-PF** (PCR primer for qRT) 5’ - CCATCGAGTTGGCGTTGATTAC - 3’ | Rui Biotech oligo |
| **AT1G61790-PR** (PCR primer for qRT) 5’ - TCCAGAAACATCATTACTCAATTCCC - 3’ | Rui Biotech oligo |
| **AT1G61790-DF** (PCR primer for RIP and qRT) 5’ - GTCTCTCATTTGACAATTTCGGGTC - 3’ | Rui Biotech oligo |
| **AT1G61790-DR** (PCR primer for RIP and qRT) 5’ - CCATTAAACATAGCAATATGTCGAAACC - 3’ | Rui Biotech oligo |
| **AT5G10700-TF** (PCR primer for RIP and qRT) 5’ - GACCAGATCGCTGCCACTGAC - 3’ | Rui Biotech oligo |
| **AT5G10700-TR** (PCR primer for RIP and qRT) 5’ - GTCGTGGTGCAATACGTTGTGC - 3’ | Rui Biotech oligo |
| **AT5G10700-DF** (PCR primer for RIP and qRT) 5’ - AACTCCCACCTCATACGAGATC - 3’ | Rui Biotech oligo |
| **AT5G10700-DR** (PCR primer for RIP and qRT) 5’ - GCCTTATCCTAAATCCCAAGTCTC - 3’ | Rui Biotech oligo |
| **AT5G10700-D2F** (PCR primer for RIP) 5’ - GGACGAGAGGGTGATCGGAAG - 3’ | Rui Biotech oligo |
| **AT5G10700-D2R** (PCR primer for RIP) 5’ - CGAAGCAGAATCTCCACTGGC - 3’ | Rui Biotech oligo |
| **AT1G24610-TF** (PCR primer for RIP and qRT) 5’ - TGCTGTGGCTGAAACACCTTG - 3’ | Rui Biotech oligo |
| **AT1G24610-TR** (PCR primer for RIP and qRT) 5’ - CCGTACTGTAATCGCTCTGTGTG - 3’ | Rui Biotech oligo |
| **AT1G24610-DF** (PCR primer for qRT) 5’ - GATCCTCTGTGTGTTGGGGTTTATC - 3’ | Rui Biotech oligo |
| **AT1G24610-DR** (PCR primer for qRT) 5’ - ACATGAATCTATGAACTAGAGAAGGGAG - 3’ | Rui Biotech oligo |
| **AT3G53130-TF** (PCR primer for RIP and qRT) 5’ - CTGATCTCCTCGGAGGTGCTC - 3’ | Rui Biotech oligo |
| **AT3G53130-TR** (PCR primer for RIP and qRT) 5’ - GCTATCGCTGGGTCGCTCAC - 3’ | Rui Biotech oligo |
| **AT3G53130-T2F** (PCR primer for RIP) 5’ - CATTGATGGAGGCAATTGTGGC - 3’ | Rui Biotech oligo |
| **AT3G53130-T2R** (PCR primer for RIP) 5’ - TGGTTGCTCCTGTGGTCATGC - 3’ | Rui Biotech oligo |
| **AT3G53130-DF** (PCR primer for RIP and qRT) 5’ - CCATTCCATATGGACCACACG - 3’ | Rui Biotech oligo |
| **AT3G53130-DR** (PCR primer for RIP and qRT) 5’ - CTACTCTCTGAGCTTTCGTTCTC - 3’ | Rui Biotech oligo |
| **Target pre-mRNA-aF** (PCR primer for RIP) 5’ - ctcttagcgaatgagctctgc - 3’ | Rui Biotech oligo |
| **Target pre-mRNA-aR** (PCR primer for RIP) 5’ - gactgctaaacgcggatacaag - 3’ | Rui Biotech oligo |
| **Target pre-mRNA-bF** (PCR primer for RIP) 5’ - cttgttttggctcgatgcttc - 3’ | Rui Biotech oligo |
| **Target pre-mRNA-bR** (PCR primer for RIP) 5’ - gggaattgaggaacaaacatgg - 3’ | Rui Biotech oligo |
| **Target pre-mRNA-cF** (PCR primer for RIP) 5’ - TGGGGAGGTCGATAAGAATC - 3’ | Rui Biotech oligo |
| **Target pre-mRNA-cR** (PCR primer for RIP) 5’ - TTGCTGCTGTTTCTGCTGTA - 3’ | Rui Biotech oligo |
| **Target pre-mRNA-PF** (PCR primer for qRT) 5’ - AGGCTCTTCAAGGAGCAAATTTG - 3’ | Rui Biotech oligo |
| **Target pre-mRNA-PR** (PCR primer for qRT) 5’ - agacaatcaaagttagagaacaaacg - 3’ | Rui Biotech oligo |
| **Target pre-mRNA-DF** (PCR primer for qRT) 5’ - cctaataatgcagcgcctcaag - 3’ | Rui Biotech oligo |
| **Target pre-mRNA-DR** (PCR primer for qRT) 5’ - TGCTTCTCCATTGTACAGTCCATTG - 3’ | Rui Biotech oligo |
| **ActinF** (PCR primer for qRT) 5’ - GGTGTCATGGTTGGTATGGGTC - 3’ | Rui Biotech oligo |
| **ActinR** (PCR primer for qRT) 5’ - CCTCTGTGAGTAGAACTGGGTGC - 3’ | Rui Biotech oligo |
